# Identification of Injury Specific Proteins in a Cell Culture Model of Traumatic Brain Injury

**DOI:** 10.1371/journal.pone.0055983

**Published:** 2013-02-07

**Authors:** Camilla Lööv, Ganna Shevchenko, Aishwarya Geeyarpuram Nadadhur, Fredrik Clausen, Lars Hillered, Magnus Wetterhall, Anna Erlandsson

**Affiliations:** 1 Department of Neuroscience, Uppsala University, Uppsala, Sweden; 2 Department of Chemistry-BMC, Analytical Chemistry, Uppsala University, Uppsala, Sweden; Georgia Health Sciences University, United States of America

## Abstract

The complicated secondary molecular and cellular mechanisms following traumatic brain injury (TBI) are still not fully understood. In the present study, we have used mass spectrometry to identify injury specific proteins in an *in vitro* model of TBI. A standardized injury was induced by scalpel cuts through a mixed cell culture of astrocytes, oligodendrocytes and neurons. Twenty-four hours after the injury, cell culture medium and whole-cell fractions were collected for analysis. We found 53 medium proteins and 46 cell fraction proteins that were specifically expressed after injury and the known function of these proteins was elucidated by an extensive literature survey. By using time-lapse microscopy and immunostainings we could link a large proportion of the proteins to specific cellular processes that occur in response to trauma; including cell death, proliferation, lamellipodia formation, axonal regeneration, actin remodeling, migration and inflammation. A high percentage of the proteins uniquely expressed in the medium after injury were actin-related proteins, which normally are situated intracellularly. We show that two of these, ezrin and moesin, are expressed by astrocytes both in the cell culture model and in mouse brain subjected to experimental TBI. Interestingly, we found many inflammation-related proteins, despite the fact that cells were present in the culture. This study contributes with important knowledge about the cellular responses after trauma and identifies several potential cell-specific biomarkers.

## Introduction

Worldwide, traumatic brain injury (TBI) is a major cause of death and disability. Despite that, there are currently no specific pharmacological agents available for neuroprotective and regenerative treatment in the neurointensive care setting. To enable such interventions in the future, a comprehensive understanding of the basic cellular and molecular secondary injury mechanisms after TBI is crucial. In addition, there is a need for sensitive and specific biomarkers of TBI with diagnostic and prognostic value [1], [2]. The complexity of the brain makes it extremely time-consuming to screen for novel treatment targets *in vivo*. Furthermore, the high diversity of cells at the injury site after TBI, including neuronal, glial, inflammatory and endothelial cells limits the possibility to follow the consequences of trauma on specific cells over time. For this purpose, *in vitro* injury models are valuable complementary tools. *In vitro* models are also useful to identify possible biomarkers and to elucidate their cellular source and function prior to further evaluation in an *in vivo* setting. It has been shown that *in vitro* models replicate *in vivo* results in close to 90% of the cases, confirming their usefulness [3]. Several different *in vitro* models of TBI have been developed including static mechanical injury such as transections, compression and barotrauma; dynamic mechanical injury, such as acceleration/deceleration and hydrodynamic injury models, and cell stretch models [4]. Despite the inherent simplifications of these *in vitro* systems, many aspects of the posttraumatic events are dependably reproduced in cultured cells, including ultrastructural changes, ionic derangements, alterations in electrophysiology, and free radical generation [5]. In the present study we have used a scratch injury model [6] with a mixed culture of primary neurons, astrocytes and oligodendrocytes, without any contaminating microglia [7], [8] to identify proteins that are specifically expressed in the cells and in the surrounding medium 24 h after trauma. The study is based on mass spectrometry (MS) analysis of the proteins in the injured and uninjured cultures. To understand how the different proteins identified by MS are involved in cellular processes after trauma, the functions of the proteins need to be carefully elucidated and to this end we thoroughly researched the available literature describing the function of the different injury specific proteins. Furthermore, we have studied cellular processes such as proliferation, cell death, migration and actin remodeling by immunostainings and time-lapse microscopy to link the injury specific proteins to events seen after trauma. An interesting finding was that several actin-associated proteins were specifically found in the medium after injury although actin itself was not. Two of these, ezrin and moesin, were of special interest since they were highly scored in the MS experiments and had previously been linked to TBI *in vivo* [9]. Ezrin and moesin are normally found intracellularly, together with radixin referred to as the Ezrin/Moesin/Radixin proteins (ERM proteins) [10]. Radixin was however not identified as an injury-specific protein in the medium, indicating that the different ERM proteins may have individual roles in addition to their known intracellular function.

## Results and Discussion

### In vitro injury demonstrates cell type specific responses to trauma

**Figure 1:**
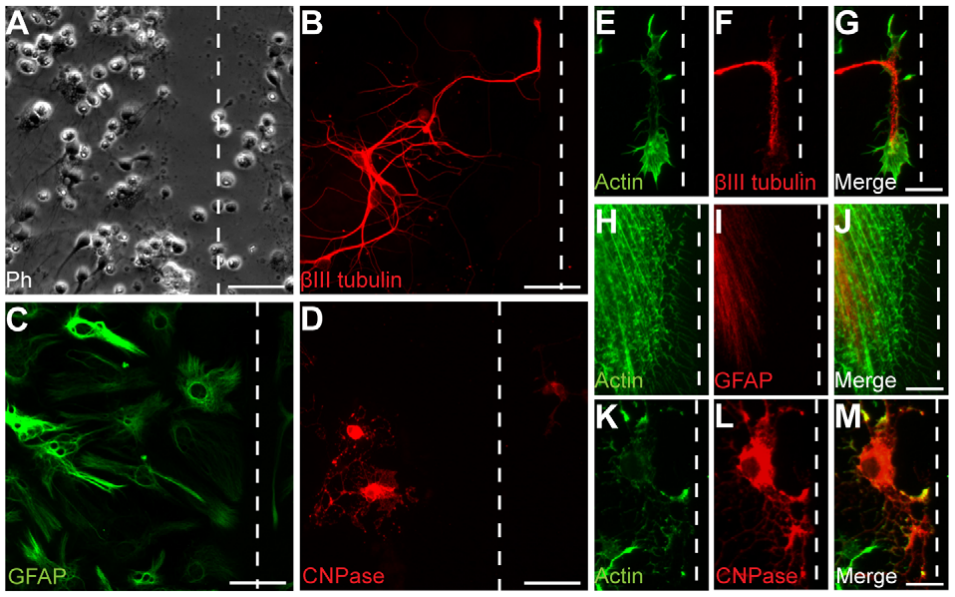
Differences in regeneration of neurons, astrocytes and oligodendrocytes after injury. (A) Phase-contrast of an injured culture. The scalpel cut create clear injury (dashed line) with surrounding cells that remain unharmed. (B–D) Displaying the individual cell types' appearance 24 h after injury. (B) The neurons have regenerated new axons towards and along the laceration, without breaching the boundary of the cut. (C) Astrocytes reach towards and along the injury, and like neurons, do not grow into the laceration. (D) Oligodendrocytes are more hesitant in their regeneration of the injury and unless in its direct vicinity, does not grow along the cut but rather avert it. (E–M) The actin patterns of the cells 24 h after injury implicates it as an important regeneratory protein in wound healing. (E–G) The outgrowing new growth cones along the injury are highly reactive for actin. (H–J) The astrocytes have extended a multitude of actin-positive lamellipodia towards and along the cut at 24 h after injury. (K–M) Twenty-four hours after injury, oligodendrocytes in direct proximity to the injury have some lamellipodia-like extensions towards the cut, although they appear more reluctant in covering the cut compared to both neurons and astrocytes. Scale bars equal 50 µm (A–D) or 10 µm (E–M) and dashed lines represent the injury.

In order to investigate the molecular and cellular changes after trauma, we used an *in vitro* scratch injury model that produces a localized and distinct injury with a clear border to surrounding uninjured cells [6]. An important advantage with this model is the high reproducibility and the distinct injury makes it possible to compare the effect on cells immediately adjacent to the injury to more distant, uninjured cells. The model is suitable for time-lapse microscopy of individual cells, immunostainings and MS analysis of proteins in the cells or the surrounding medium. Due to its simplicity, the scratch model has limitations in reflecting the complexity of the injured brain, but is a good tool to screen for possible treatment targets and biomarkers. In short, E14 mouse cortices, first grown into neurospheres, where seeded as single cells on glass coverslips and differentiated for 8 days into neurons, astrocytes and oligodendrocytes [7]. The cell layer was then injured by a scalpel cut 20 times in two directions (Figure 1A). After injury, the plates were incubated for 24 h, fixed and stained by immunocytochemistry against specific markers for neurons (βIII tubulin, Figure 1B), astrocytes (GFAP, Figure 1C) and oligodendrocytes (CNPase, Figure 1D). The major advantage with this cell culture system, compared to a traditional neuron-glia co-culture (not based on differentiated neural stem cells) is that the cells are cultured together in the same kind of medium throughout the experiment, thereby excluding changes in protein expression as a result of the medium change. Moreover, the neural stem cell based culture system enables co-culturing of neurons, astrocytes and oligodendrocytes without any contaminating microglia and other non-neural cells.

Time-lapse microscopy demonstrated that in the first minutes after injury, the extensions of the neurons and glia closest to the cut degenerate before they start to regenerate towards the area again. The cut itself does not kill the cells if not cut directly through or close to their nuclei (Video S1). The time-lapse experiments, in addition to immunostainings with βIII tubulin and phalloidin (to identify the actin of the axonal growth cones) clearly show that the regenerating neurites grow along the scratches (Figure 1E–G). Furthermore, our time-lapse movies demonstrate that neurons, identified by their oval cell bodies and distinct axons, actively migrate towards and along the injury (Video S1). Similarly to neurons, the astrocytes (identified by their phenotype of an egg “sunny side up”) regenerate their processes quickly after trauma, but contrary to neurons, the astrocytes do not migrate in response to injury (Video S1). Immunostainings against GFAP and phalloidin show that astrocytic lamellipodia reach towards the injury, but very seldom cross it (Figure 1H–J). Oligodendrocytes do not display the same reaching pattern as the other cell types do towards the injury. Several cells show diffuse actin patterns and only a few show actin structures at the ends of their extensions and their regeneration towards the scratch is less pronounced compared to astrocytes (Figure 1K–M).

### Mass Spectrometry identifies proteins that are specifically expressed after injury

To search for possible biomarkers of neural trauma, we sought to identify which extracellular and intracellular proteins that were present early after injury by using MS. The focus was set on proteins that were specifically expressed in the cells or the culture medium after injury. The cells were plated and injured (as described above) and incubated for 24 h. The medium was collected from both uninjured and injured cultures, centrifuged to clear any cells and debris, and analyzed by nanoLC-MS/MS. The cells were mechanically removed and saved for separate MS analysis after removal of any remaining liquid. A standardized amount of 35 µg protein was analyzed from each sample. All keratins were removed from the list due to a risk of contamination from laboratory equipment and personnel. To verify proteins that are universally present after injury, all samples were run from two independent cell cultures and compared in order to find overlapping proteins during both MS analyses. The medium samples had a total of 165 overlapping proteins in uninjured cultures (Figure S1A) and 155 proteins in the injured cultures (Figure S1B). In the cell fractions, 323 overlapping proteins were found in the uninjured cells (Figure S1C) and 275 in the injured cells (Figure S1D). Since our aim was to identify proteins that were unique to injury, both the medium and the cell fractions were compared and all overlapping proteins were concidered to be generally expressed and are not dicussed further in this article. The use of very strict filtering criterias resulted in 53 proteins found in the medium after injury as compared to uninjured controls (Table 1 and Table S1). The cell fraction contained 46 uniquely produced proteins after injury (Table 2 and Table S2). We searched National Institutes of Health's US Library of Medicine (PubMed) for previous reports describing the function/s of the injury specific proteins and the staggering amount of data was sorted by certain topics: Actin; Migration/Motility/Chemotaxis; Neurite/Growth cones; Proliferation/Differentiation/Cell death/Survival; Neurological disease/degeneration/TBI; Engulfment/Degradation; Immune response; ER/Golgi/Secretion/Energy metabolism and Scar formation/Reactive gliosis. Table 1 and 2 show to which group/s the respective proteins discussed in the text belong. In addition, all proteins and the corresponding references are presented in Supporting Table 1 and 2. The proteins are presented in the order of highest to lowest protein score, with a cut off at 26, where a higher protein score represents a more abundant protein.

**Table 1:** Previously shown functions for the proteins found in medium exclusively after injury.

Actin	Migration/Motility/Chemotaxis	Neurite/Growth cones	Proliferation/Differentiation/Cell death/Survival	Engulfment/Degradation	Immune response	Neurological disease/degeneration/TBI
Fructose-bisphosphate aldolase A	Nucleoside diphosphate kinase A	Fructose-bisphosphate aldolase A	14-3-3 protein γ	Ezrin	Fructose-bisphosphate aldolase A	14-3-3 protein γ
Ezrin	Ezrin	Nucleoside diphosphate kinase A	Histone H1.2	Moesin	Ezrin	Fructose-bisphosphate aldolase A
Moesin	Legumain	Calponin-3	Fructose-bisphosphate aldolase A	Actin-related protein 2	Legumain	Rab GDP dissociation inhibitor α
Cofilin-1	L-lactate dehydrogenase A chain	Protein NDRG2	Nucleoside diphosphate kinase A	Lysosome-associated membrane glycoprotein 1	Peroxiredoxin-1	Nucleoside diphosphate kinase A
Four and a half LIM domains protein 1	Moesin	Actin-related protein 2	Ezrin	Myristoylated alanine-rich C-kinase substrate	Moesin	Ezrin
Rho GDP-dissociation inhibitor 1	Rho GDP-dissociation inhibitor 1	Myristoylated alanine-rich C-kinase substrate	Fatty acid synthase	F-box only protein 2	Cofilin-1	Fatty acid synthase
Calponin-3	Calponin-3	Destrin	Latexin	Destrin	Nascent polypeptide-associated complex subunit α	Peroxiredoxin-1
N(G),N(G)-dimethylarginine dimethylaminohydrolase 1	N(G),N(G)-dimethylarginine dimethylaminohydrolase 1		Moesin	WD repeat-containing protein 1	Protein DJ-1	Latexin
Protein NDRG2	Actin-related protein 2		Histone H1.1		Lysosome-associated membrane glycoprotein 1	Moesin
Actin-related protein 2	Myristoylated alanine-rich C-kinase substrate		Nascent polypeptide-associated complex subunit α		Peptidyl-prolyl cis-trans isomerase FKBP1A	Cofilin-1
Myotrophin	Glutamine synthetase		Rho GDP-dissociation inhibitor 1		Myristoylated alanine-rich C-kinase substrate	Nascent polypeptide-associated complex subunit α
Myristoylated alanine-rich C-kinase substrate	Thymosin β-4		Protein DJ-1		Thymosin β-4	Rho GDP-dissociation inhibitor 1
Thymosin β-4	Destrin		Calponin-3			Protein DJ-1
Destrin	WD repeat-containing protein 1		10-formyltetrahydrofolate dehydrogenase			Calponin-3
WD repeat-containing protein 1	Follistatin-related protein 1		N(G),N(G)-dimethylarginine dimethylaminohydrolase 1			6-phosphogluconate dehydrogenase, decarboxylating
			Probable ATP-dependent RNA helicase DDX17			10-formyltetrahydrofolate dehydrogenase
			Protein NDRG2			Protein NDRG2
			Actin-related protein 2			Proteasome subunit α type-5
			Lysosome-associated membrane glycoprotein 1			Histidine triad nucleotide-binding protein 1
			Proteasome subunit α type-5			Peptidyl-prolyl cis-trans isomerase FKBP1A
			Peptidyl-prolyl cis-trans iso-merase FKBP1A			Small ubiquitin-related modifier 2
			Myotrophin			Microtubule-associated protein tau
			Small ubiquitin-related modifier 2			Myristoylated alanine-rich C-kinase substrate
			Myristoylated alanine-rich C-kinase substrate			F-box only protein 2
			F-box only protein 2			Insulin-like growth factor-binding protein 2
			Insulin-like growth factor-binding protein 2			Glutamine synthetase
			Glutamine synthetase			Thymosin β-4
			Thymosin β-4			60S ribosomal protein L5
			Destrin			Destrin
			Farnesyl pyrophosphate synthase			Follistatin-related protein 1
			WD repeat-containing protein 1			T-complex protein 1 subunit γ
			Follistatin-related protein 1			

**Table 2:** Previously shown functions for the proteins found in cells exclusively after injury.

Actin	Migration/Motility/Chemotaxis	Neurite/Growth cones	Proliferation/Differentiation/Cell death/Survival	Engulfment/Degradation	Immune response	Neurological disease/degeneration/TBI
γ-adducin	Tubulin β-2B chain	Tubulin β-2B chain	Tubulin β-2B chain	V-type proton ATPase 116 kDa subunit a isoform 1	Heat shock-related 70 kDa protein 2	Tubulin β-2B chain
Fructose-bisphosphate aldolase A	Dihydropyrimidinase-related protein 1	Dihydropyrimidinase-related protein 1	Heat shock-related 70 kDa protein 2	Sarcoplasmic/endoplasmic reticulum calcium ATPase 2	UDP-glucose:glycoprotein glucosyltransferase 1	Heat shock-related 70 kDa protein 2
Gelsolin	L-lactate dehydrogenase A chain	V-type proton ATPase 116 kDa subunit a isoform 1	Dihydropyrimidinase-related protein 1	Gelsolin	V-type proton ATPase 116 kDa subunit a isoform 1	Dihydropyrimidinase-related protein 1
α-adducin	γ-adducin	Contactin-1	10-formyltetrahydrofolate dehydrogenase	Lysosome-associated membrane glycoprotein 2	Sodium- and chloride-dependent GABA transporter 1	10-formyltetrahydrofolate dehydrogenase
	Gelsolin	γ-adducin	Contactin-1	V-type proton ATPase subunit d 1	Myelin proteolipid protein	Sodium- and chloride-dependent GABA transporter 1
		Fructose-bisphosphate aldolase A	L-lactate dehydrogenase A chain		RuvB-like 2	Myelin proteolipid protein
		Gelsolin	Fructose-bisphosphate aldolase A		Gelsolin	Contactin-1
			Carboxypeptidase E		Lysosome-associated membrane glycoprotein 2	Fructose-bisphosphate aldolase A
			Sarcoplasmic/endoplasmic reticulum calcium ATPase 2		Thioredoxin-related transmembrane protein 2	Carboxypeptidase E
			Gelsolin		RuvB-like 1	Gelsolin
			Glycine amidino-transferase, mitochondrial		Disintegrin and metalloproteinase domain-containing protein 28	Glycine amidinotransferase, mitochondrial
			2-oxoglutarate dehydrogenase, mitochondrial			2-oxoglutarate dehydrogenase, mitochondrial
			Hexokinase-1			Hexokinase-1
			2′,3′-cyclic-nucleotide 3′-phosphodiesterase			Lysosome-associated membrane glycoprotein 2
			Thioredoxin-related transmembrane protein 2			2′,3′-cyclic-nucleotide 3′-phosphodiesterase
			40S ribosomal protein S3			Ferritin light chain 1
						Probable ATP-dependent RNA helicase DDX5
						Nucleolin

### Actin related proteins are found in the medium after injury

Actin is one of the most abundant protein in a cell and functions in cell structure, migration, proliferation, cell signaling and phagocytosis [11]. The surprising finding in our MS data was that several of the highest scored proteins found after injury in the medium fraction had actin interacting properties, although they are thought of as intracellular proteins. Out of the 53 proteins specifically found in the medium after injury, 15 were associated with actin (28%). However, we did not find actin itself in any of our medium fractions, making it possible that the actin related proteins are freely secreted into the extracellular space as a response to injury. In contrast to the medium fraction, the injured cells had a lower representation of actin-related proteins after injury (4 of 46, 9%). The appearance of extracellular proteins after injury that in normal conditions are situated exclusively intracellularly, points to their potential use as biomarkers after injury.

**Figure 2:**
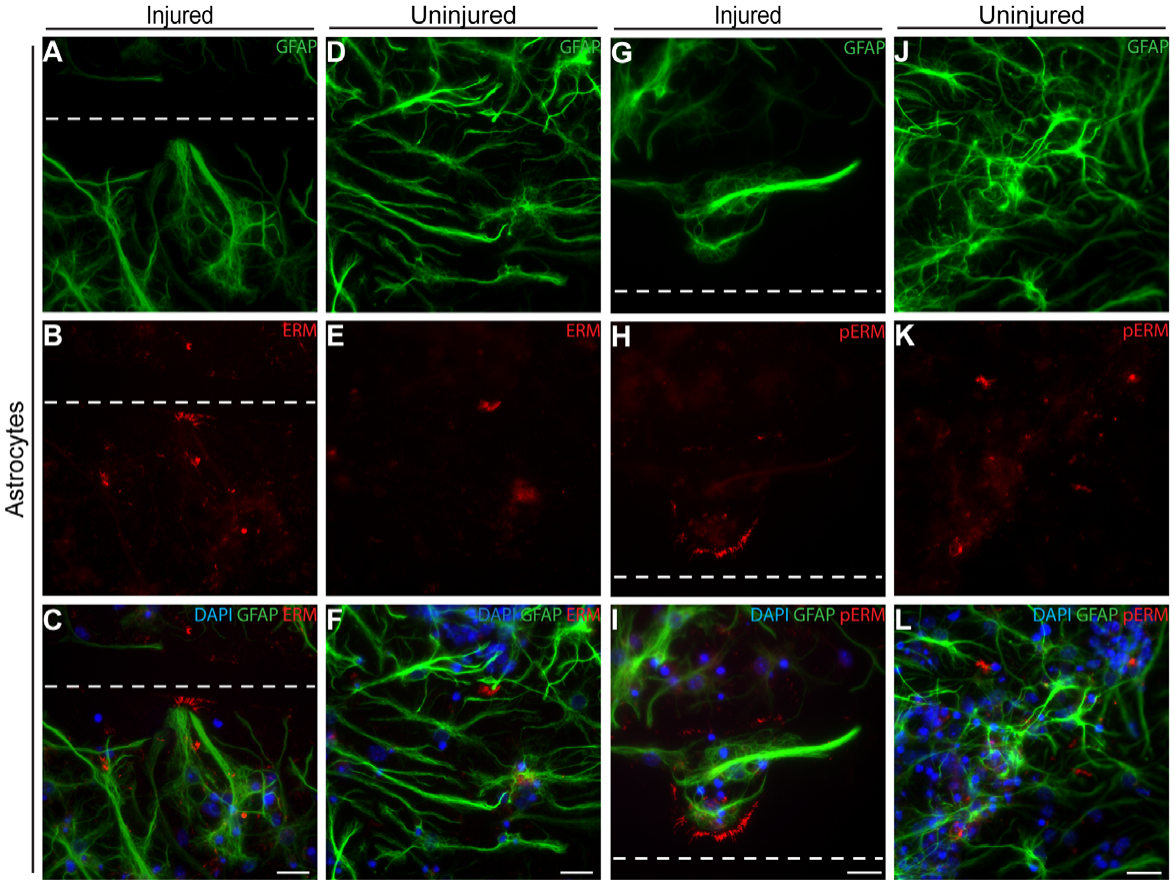
Astrocytes are most likely the source of the ezrin and moesin found specifically in medium after injury. (A–F) The ERM is expressed by astrocytes in a fingerlike pattern at 24 h post-injury (A–C) in comparison to the disorganized expression mostly seen in uninjured cultures (D–F). (G–L) Stainings with antibodies specifically against the active, phosphorylated form of ERM reveal the intracellular ERM expressed by astrocytes appear to be phosphorylated as it co-localizes with the expression patterns of ERM after injury (G–I) as well as the occasional patches of ERM seen in uninjured cultures (J–L). Scale bars equal 50 µm and dashed lines represent the injury.

The two actin associated proteins ezrin and moesin are of specific interest since their expression is known to be up-regulated following brain trauma *in vivo* [9]. In order to elucidate which cells produced the ERM proteins and their active phosphorylated form (pERM) we performed immunostainings with specific antibodies against the ERM proteins and pERM in combination with the cell type markers: GFAP (astrocytes) (Figure 2), βIII tubulin (neurons; Figure S2) or CNPase (oligodendrocytes; Figure S2). Before injury, most confluent astrocytes had no specific pattern in their ERM expression, whereas the injured astrocytes displayed a distinct finger-like expression in the injury reaching lamellipodia (Figure 2A–F), which co-localized with its active, phosphorylated form (Figure 2G–L). On the contrary, neurons and oligodendrocytes barley expressed any ERM or pERM and no change in protein expression was found in response to injury (Figure S2). This demonstrates that astrocytes are the most likely source of the extracellular ezrin and moesin found in the medium after injury.

**Figure 3:**
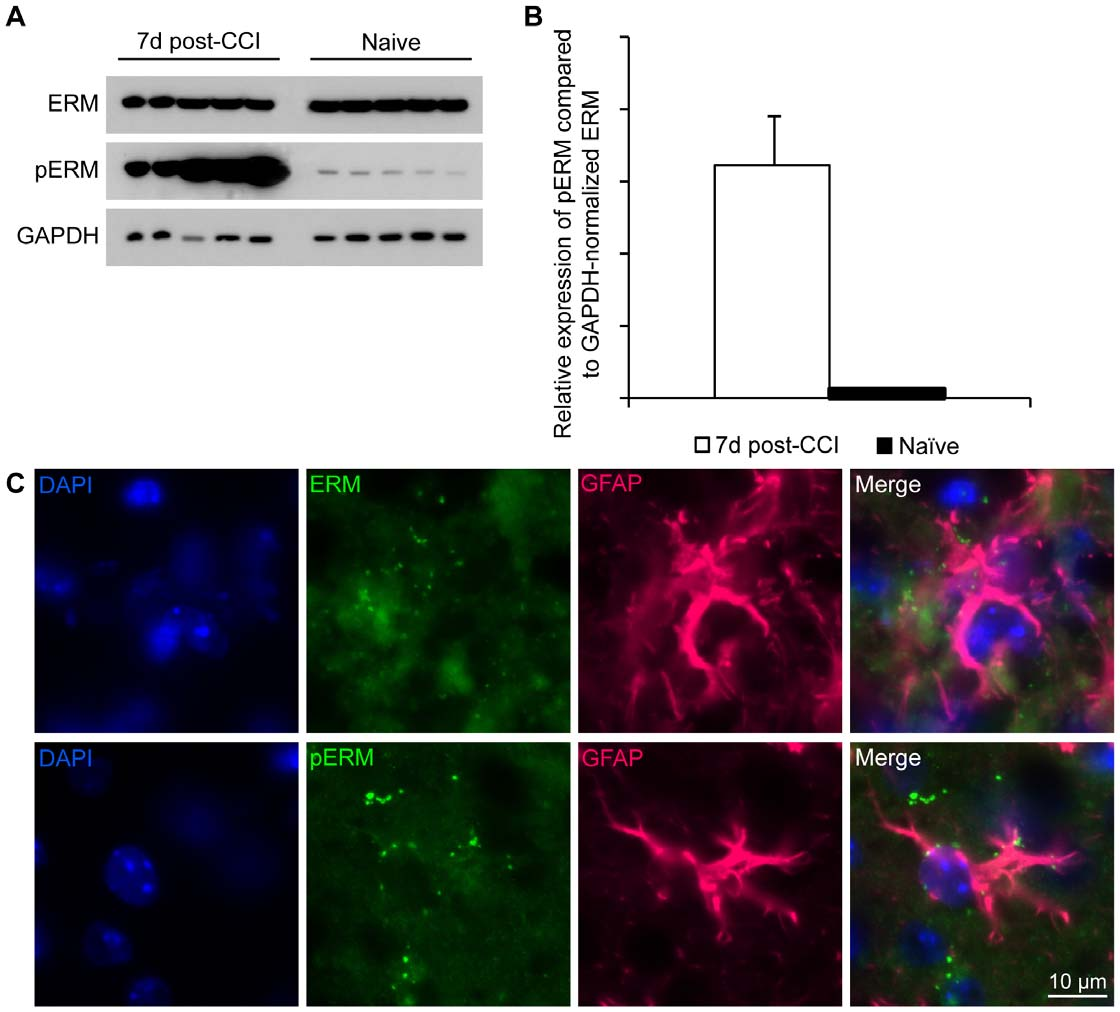
Phosphorylation of ERM is greatly enhanced after TBI in mice. (A) Western blot analysis of cell lysates from cerebral cortex of mice that had been subjected to TBI (n = 5, lanes to the left) and uninjured controls (n = 5, lanes to the right) show that, although ERM expression remain stable post-CCI, injury induce activation of ERM by phosphorylation. (B) There is a 25-fold increase in pERM after injury as compared to naïve animals. Bars represent the average relative expression of pERM as compared to GAPDH normalized ERM. Error bars represent SEM. (C) Similarly to the in vitro injury, ERM and pERM is expressed in lamellipodia-like extensions in astrocytes reaching towards the injury. Representative images of ERM and pERM expression seen in brain slices from mice 7 days post-CCI.

In order to confirm that ezrin and moesin were present following TBI *in vivo*, adult mice were subjected to controlled cortical impact (CCI) and the injured and uninjured cortical brain tissue was investigated by Western blot analysis and immunofluorescence stainings. The Western blot analysis of total brain lysates from five injured mice and five uninjured controls showed that the ERM proteins were expressed both in the uninjured and injured brain (Figure 3A). Interestingly, there was a 25-fold increase in the proportion of activated, phosphorylated protein, pERM, after TBI (Figure 3B). Our immunofluorescence stainings demonstrated that in line with our results from the *in vitro* experiments, astrocytes express ERM and pERM in their processes and in vesicle like structures after CCI (Figure 3C).

Actin is considered a house-keeping protein, and though it was not found in the medium, we wanted to study whether the injury impacted the levels of total actin and not only the proteins interacting it. Western blot analyses of injured and uninjured cell culture lysates were performed (*n* = 6). We found that there were no differences in the injured/uninjured actin ratio (0.998±0.123, mean ± SEM), and neither were there any differences in the injured/uninjured ratio of two other known house-keeping proteins, GAPDH and Histone H2B (data not shown). These results clearly show that the total actin expression is stable in the cells, even though several of the processes that are induced after injury, such as cell migration, neurite regeneration and lamellipodia formation, involves extensive actin remodeling.

### Injury specific proteins involved in the regulation of cell migration and axonal regeneration

**Figure 4:**
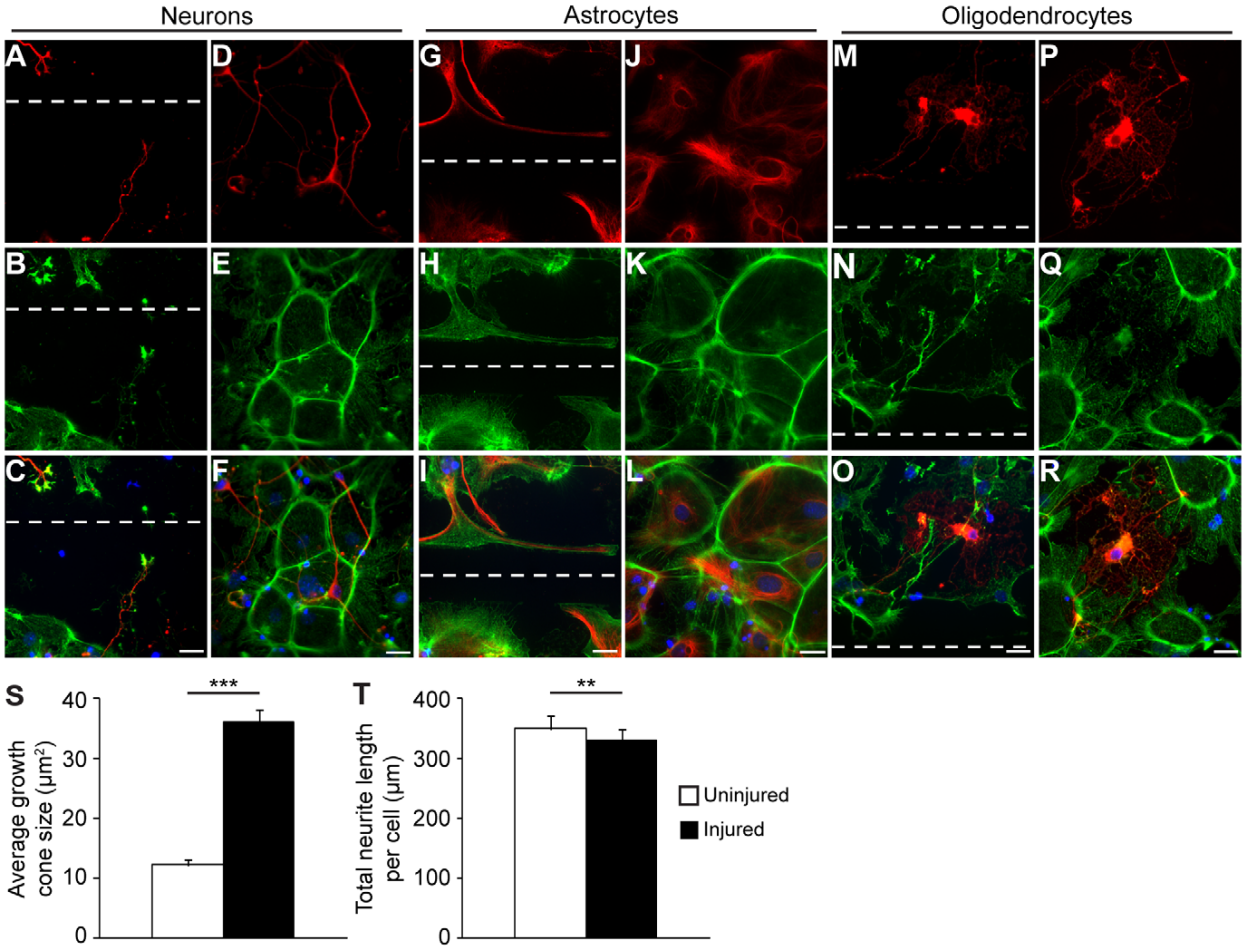
Injury results in intracellular reorganization of actin and changes the neurite characteristics. After injury, the neurons start to generate new and bigger actin-positive growth cones, which stretch towards the cut (A–C) compared to the neurons in the uninjured culture, which have fewer and smaller growth cones (D–F). The astrocytes develop an abundance of actin-rich lamellipodia that stretch towards and along the injury (G–I). The uninjured astrocytes, on the other hand, express actin in a tile-like pattern (J–L), which is disrupted by the injury. Oligodendrocytic actin expression is diffuse and remains largely unchanged after injury (M–O) compared to uninjured controls (P–R). Scale bars equal 50 µm and dashed lines represent the cut. (S) There is a 3-fold increase in growth cone size after injury. The sizes of 30 growth cones in injured and uninjured cultures of three independent experiments were measured and the average area per growth cone was calculated. Error bars represent SEM. (T) Injury leads to shorter neurites. The total neurite lengths of 50 neurons in injured and uninjured cultures of three independent cultures were measured and an average of the total length of the neurites per cell was calculated. Error bars represent SEM.

Time-lapse imaging and neuronal stainings show a clear renewal of neurites after injury (Figure 1E–G, Video S1). In order to compare the percentage of neurites with growth cones along the injury and in uninjured controls, we performed staining with βIII tubulin and phalloidin (Figure 4A–F). The total number of neurites and the number of neurites with growth cones within each field at 20X magnification were counted and the percentage of growth cone positive neurites calculated (*n* = 3, 10 fields per slide). There was a significant increase in the percentage of neurites with growth cones around the injury compared to uninjured controls. In the injured area 100.0±0% (mean ± SEM) of the neurons had growth cones while the percentage in uninjured control cultures was 71.1±4.4% (mean ± SEM) (*p*<0.001, t test). Differences were not only noticeable in the numbers of growth cones but the growth cones close to the injury were also much larger (Figure 1E–G, Figure 4A–C, Figure 4T) than in the uninjured cultures (Figure 4D–F, Figure 4T). By using the marker Gap43, we determined the size of the growth cones in injured and uninjured cultures by measuring their areas with the AxioVision outline tool (*n* = 4, 30 growth cones in uninjured respective injured cultures). Our result showed that there was a 3-fold increase in growth cone size in the injured compared to the uninjured cultures (Figure 4T). To clarify if the neurite length was also affected by the injury, the neurons were stained for βIII tubulin and the neurites of 50 cells in both uninjured and injured cultures were measured in three independent cultures. The total neurite length per cell was significantly shorter after injury compared to uninjured cells (*p*<0.01, t test) (Figure 3S). On average, the total length per cell was 174.7±11.5 µm (mean ± SEM) in the uninjured cultures and 136.7±8.4 µm (mean ± SEM) in the injured neurons. The shorter neurite length after injury could be due to that the neurites had not fully recovered from being transected by 24 h after injury. The average number of neurites per cell was unchanged in injured compared to uninjured neurons, 4.62±0.23 (mean ± SEM) and 4.91±0.25 (mean ± SEM) respectively, indicating that the general morphology remained unchanged post-injury.

Neuronal migration was induced in a chemotactic-like manner towards the injury (Video S1) compared to the undirectional migration observed in the uninjured cultures (Video S2). In contrast to neurons, both astrocytes and oligodendrocytes were quite immobile. However, after injury the astrocytes extend countless lamellipodia towards the injury but, as mentioned above, rather grow along than into the injury site (Figure 1H–J and Figure 4G–I).

The injury clearly promoted growth cone formation as well as directed migration and MS analysis of the medium showed that 7 of 53 (13%) uniquely expressed proteins had previously been associated with neurites and growth cones and 15 of 53 proteins (28%) with migration. Of the proteins found uniquely after injury in the cell fraction, 7 out of the 46 proteins (15%) have shown effects on neurite regeneration and 5 of 46 (11%) on migration. In the medium, nucleoside diphosphate kinase A (NDKA) was found to be of special interest since it has been shown to positively stimulate neurite outgrowth [12]. Two other proteins found in the medium, actin-related protein 2 (ARP2), a vital part of the ARP2/3 complex, and myristoylated alanine-rich C-kinase substrate (MARCS) have been shown to be important for lamellipodia formation [13], [14]. ARP2 is, in addition, also important for neurite outgrowth [14]. The protein N(G),N(G)-dimethylarginine dimethylaminohydrolase 1 (DDAH1) increases cell motility by affecting RhoA [15], [16]. In contrast, the highly scored Rho-GDI dissociation inhibitor 1 (GDIR1), also found uniquely in the medium after injury, has been shown to have an inhibitory function on migration [17], [18]. Our cell culture system includes neurons, astrocytes and oligodendrocytes and the proteins found in the medium after injury might affect each cell type differently, as is the case of the chemotactic movement of neurons towards the injury after trauma compared to the relative immobility of glial cells (Video S1 and S2). It is known that neurons in particular are partial to topographical cues, such as for example the rifts created by the scalpel in this case, but also that extracellular biochemical cues in concert with topographical have a synergistic effect [19]. As neurons are attracted to the injury from afar, the initial migration towards the cut is most likely due to extracellular signals, but to what extent the topography of the rifts or the orientation/signaling from astrocytes in its vicinity is responsible for the neurons staying there, remains to be elucidated.

### Cell death, survival and subsequent proliferation in the face of injury

Fifty-eight percent of the unique proteins in the medium (31 of 53 proteins) and 35% (16 of 46 proteins) of the cell fraction proteins have previously been linked to cell death, survival and/or proliferation. Our time-lapse imaging shows that cells with a neuronal phenotype divide more frequently after injury (Video S1) compared to uninjured cell cultures (Video S2). To confirm this, we quantified the percentage of dividing cells that expressed specific markers for neurons, astrocytes or oligodendrocytes. Parallel cultures were injured and incubated for 24 h. Following fixation, the cultures were stained with specific antibodies against the proliferation marker Ki67 and the cell type markers βIII tubulin, GFAP or CNPase (*n* = 3). In line with the time-lapse experiments, we found that injury induced proliferation predominantly of βIII tubulin positive cells. Along the injury, more than half of the dividing cells (54±4%, mean ± SEM) were neurons/neuroblasts. In addition, we found that some of the Ki67 positive cells (38±7%, mean ± SEM) were astrocytes, but none (0±0%, mean ± SEM) of the dividing cells were oligodendrocytes. Interestingly, there was no difference in the percentage of dividing neurons/neuroblasts close (54±4%, mean ± SEM) or farther (55±4%, mean ± SEM) from the injury, indicating that the increase in neuronal proliferation may be dependent on exogenous, soluble factors spreading in the medium. It is important to note that most neurons in our culture system, although expressing neuronal markers such as βIII, are not fully mature.

The MS analysis shows that proteins that are involved both in cell survival and proteins that either induce or reduce proliferation could be found in the medium after injury including 14-3-3gamma (1433G), latexin, nascent polypeptide-associated complex subunit alpha (NACAM), protein NDRG2, MARCS, tymosin beta-4 (TYB4) and insulin-like growth factor-binding protein 2 (IBP2). Among the more interesting proteins with a high proteins score was 1433G, which has been found to protect astrocytes from post-ischemic cell death by binding to the activated pro-apoptotic protein Bad [20].

### Proteins involved in cell clearance and immune responses after injury

Trauma leads to a considerable amount of dead cells and inflammatory responses are now considered to be an important component of TBI [21], [22]. A rapid clearance of dead cells is vital to reduce secondary necrosis and leakage of cytotoxic material that would augment the inflammatory response. We have previously shown that astrocytes have the ability to engulf whole, dead cells following injury both *in vitro* and *in vivo* [6]. In this study we found unique proteins, which previously have been linked to engulfment (phagocytosis, macropinocytosis and autophagy) or degradation in both the medium and the cell fraction after injury. In the medium 9 of the 53 proteins (17%) and in the cells 5 of 46 proteins (11%) have previously been shown to be associated with either engulfment and/or degradation. Twelve of 53 proteins (23%) of the medium proteins and 11 of 46 proteins (24%) in the cell fraction were found to be associated to the immune response although no inflammatory cells were present in the cell culture.

The proteins ezrin and moesin (often studied together as the ERM proteins) were highly scored out of the medium proteins after injury. Intracellularly, active ezrin stimulate filamentous actin around the phagosomes and is important for proper lysosomal fusion [23]. Our immunostainings, using specific antibodies against ERM and pERM, show that in addition to their presence in astrocytic lamellipodia, ERM proteins are strongly expressed around engulfed, dead cells both *in vitro* (Figure S3A) and *in vivo* (Figure S3B). Two other proteins involved in phagosomal maturation were lamp1 and lamp2, found in the medium respective cellular fraction. It has been shown that double-mutants of lamp1 and lamp2 have impaired degradation due to failed fusion of lysosomes and phagosomes [24].

During phagocytosis membrane is consumed and when the membrane stores are exhausted there is an increase in fatty acid synthase [25], an enzyme involved in membrane synthesis, which was specifically found in the medium after injury. Several other proteins linked to engulfment were also found in the cells, including gelsolin, which is known to be important for the formation of the phagocytic cup [26], and V-type proton ATPase 116 kDa subunit a isoform 1, which has been shown to play a role in the degradation of dead cells in microglia [27]. There are no microglia present in our cell cultures suggesting that the identified proteins are expressed by engulfing astrocytes.

In previous studies it has been suggested, but not confirmed, that astrocytes may act as antigen presenting cells following injury, but whether they are capable of activating T-cells or not remains controversial [28], [29]. Interestingly, one of the highest scored injury specific proteins found in medium, legumain, is part of the antigen processing [30]. Another antigen presentation protein found was UDP-glucose:glycoprotein glucosyltransferase 1, a folding sensor involved in MHC class I surface exposure [31]. Furthermore, peroxiredoxin-1, which was specifically expressed in the medium following injury, has been suggested as a danger signal due to its ability to induce TNFα and IL-6 secretion, as well as maturation of dendritic cells [32]. Because of the lack of inflammatory cells in our culture system, the results from our MS analysis probably reflects the early changes in protein expression after TBI. These proteins may be involved in the activation and recruitment of professional inflammatory cells to the injury zone as previous studies of TBI in rats show that T lymphocyte and neutrophil infiltration peak at 24 h post-injury. Macrophages and microglia are activated much later and peak in number at 7 days post-injury [33].

### Many proteins have been linked to neurodegeneration and CNS disorders

Of all injury specific proteins found, 31 of 53 (58%) in the medium fractions and 18 of 46 (39%) in the cell fractions had previously been linked to TBI, CNS disorders or neurodegeneration. Among these proteins are both proteins that are involved in destructive processes such as cell death or oxidative stress and proteins that are involved in regenerative processes such as proliferation, survival, neurite regeneration and motility. None of the proteins in the cell fraction had previous TBI associations, but several of the proteins had been linked to ischemia, multiple sclerosis, Alzheimer's (AD), Parkinson's or Huntington's diseases. Hence, the proteins identified uniquely after injury previously linked to TBI were all found in the medium fraction. Of the highly scored proteins, ezrin and moesin were especially interesting since they have been shown to be greatly enhanced after cryogenic brain injury [9]. Our results show that ERM and pERM are expressed by astrocytes, especially adjacent to the injury both *in vitro* and *in vivo* (Figure 2 and Figure 3), but ERM and pERM is also found around engulfed dead cells within astrocytes in the cell culture and in the injured brain (Figure S3). The fact that ezrin and moesin are specifically expressed in the medium following injury indicates that they might have an unknown extracellular function and that the individual ERM proteins play different roles separate from their otherwise known functions. Two other actin-associated proteins that have been linked to TBI, calponin and cofilin-1 were both found in the medium after injury. Calponin has been shown to lead to endothelial contraction and hypoperfusion after TBI [34]. Cofilin-1 is involved in the reorganization of actin in dendritic spines and it has been shown to be up-regulated and activated 24 h after TBI [35]. Peptidyl-prolyl cis-trans isomerase FKBP1A (FKBP1A) is also involved in this process and was found specifically in medium after injury. Neutralization of FKB1A 1 h post-TBI prevented the activation of cofilin-1 and thereby stabilized the dendritic spines and preserved the integrity of neuronal connections in the injured brain [35], [36]. TYB4 has been used as a successful TBI treatment in rats. Single injections of the protein improved sensorimotor function and spatial learning and enhanced angiogenesis and neurogenesis in the injured cortex and hippocampus [37].

It has been demonstrated that there is an epidemiological association between traumatic brain injury and the development of AD later in life, although the cellular mechanism behind this remains unclear [38]. It is therefore highly interesting that we found many proteins in our *in vitro* model of TBI that previously have been linked to AD. One of these proteins, microtubule-associated protein tau (TAU) has also been found to be up-regulated after trauma. In chronic traumatic encephalopathy, a progressive neurodeterioration seen in for example boxers, there are extensive TAU positive neurofibrills throughout the brain [39] and transgenic AD mice also exhibit TAU immunoreactivity after TBI [40].

## Conclusions

This study contributes with important information about the cellular and molecular mechanisms after experimental TBI and identifies several potential biomarkers, for both regeneration and degeneration, to be studied further *in vivo*. Interestingly, many of the proteins specifically found in the medium after injury were actin-related proteins, normally situated intracellularly. Two of these, ezrin and moesin, were among the highest scored proteins. Importantly, we have also confirmed the presence of activated ezrin and moesin after TBI *in vivo*. Western blot analysis of brain lysates from five injured mice and five uninjured controls showed 25-fold increase of activated, phosphorylated ERM after TBI. Our immunostainings of cell cultures and brain slices show that astrocytes are the most likely source for these proteins and that the ERM proteins are specifically expressed in astrocytic lamellipodia and surrounding engulfed dead cells.

## Materials and Methods

### Animals

All animal experiments were done in full compliance with Swedish animal welfare legislation and the study was specifically approved by Uppsala Animal Ethics Committee, Uppsala, Sweden (Permit number: C 234/8) before the study started. The mice were housed at 24°C in 12 h light/dark cycles with access to food and water ad libitum.

### Traumatic Brain Injury In Vivo

Adult C57/BL6 mice (20–25 g) was subjected to controlled cortical impact (CCI) after isoflurane (4% in air) induced anesthesia. The mice were attached to a stereotaxic frame and anesthesia was maintained by 1.3% isoflurane in mixture with 70% nitrous oxide and 30% oxygen through a nose cone. Core body temperature was kept at 37±0.3°C by a heating pad connected to a rectal probe (CMA150, CMA Microdialysis AB, Kista, Sweden). A midline incision of the scalp was made after an s.c. injection of bupivacaine (Marcain®, AstraZeneca, Sweden). A rectangular craniotomy, 4×4 mm was created 1 mm posterior to bregma over the right parietal cortex. The injury was produced using a CCI-device (VCU Biomedical Engineering Facility, Richmond, Virginia, USA) with a 2.5 mm diameter piston, set at a compression depth of 0.5 mm and a speed of 3 m/s. After the injury the wound was closed up with interrupted sutures and the mouse moved to a heated cage to wake up. When fully awake the mouse was returned to its home cage. Animals were sacrificed 7 days post injury by a pentobarbital sodium overdose (600 mg/kg). For the immunostaining experiments, the mice were perfused with isotonic saline solution followed by 4% phosphate-buffered formaldehyde (Histolab AB, Gothenburg, Sweden). The brains were frozen and cryo-sectioned coronally to a thickness of 14 µm.

### Neural cell cultures

Cerebral cortices from C57/BL6 E14 mouse embryos were grown into neurospheres, separated into single cells and seeded on poly-L-ornithine and laminin coated cover-slips for differentiation, as previously described [6]. The cells were differentiated for 8 days before the experiments were performed.

### Cell cultures for MS analysis

To avoid any contamination of proteins from the serum free supplement used (B-27 Supplement, Invitogen), the cells were washed twice with B27-free medium before adding a total volume of 2 ml medium without B-27 per well. The injury was induced with a scalpel cut 20 times through the differentiated neural culture, 10 times perpendicularly in either direction, approximately 2 mm apart. Injured cultures and control cultures were then incubated for 24 h in 37°C, 5% CO_2_ in humidified air. The medium was collected from both uninjured and injured cultures, centrifuged in 4°C, for 10 min at 10,000× g to clear any cells and debris, and stored in new low bind Eppendorf tubes in −70°C until analyzed. The adherent cells were mechanically removed with a cell lifter and saved separately in low bind Eppendorf tubes after removal of any remaining liquid by centrifugation in 4°C, for 10 min at 10,000× g. All experiments were carried out in independent duplicates.

### Antibodies and stainings

Primary antibodies used were against the following targets: Mouse tubulin beta III isoform (βIII tubulin, 1∶200 Chemicon), Rabbit Glial Fibrillary Acidic Protein (GFAP, 1∶400, DakoCytomation), Mouse antibody against GFAP (1∶400, Sigma Aldrich), mouse 2′,3′-cyclic nucleotide 3′-phosphodiesterase (CNPase, 1∶500, Sigma), rat Ki67 (1∶200, DakoCytomation), rabbit Ezrin/Moesin/Radixin antibody (ERM, 1∶200, Abcam), rabbit phospho-Ezrin (Thr567)/Radixin (Thr564)/Moesin (Thr558) (pERM, 1∶25, Cell Signaling) and rabbit growth associated protein 43 (Gap43, 1∶100, Chemicon). Secondary antibodies (IgG) used were: AlexaFluor 488, AlexaFluor 555 or AlexaFluor 647-conjugated antibody against rabbit or mouse (1∶400, Molecular Probes), anti-rat conjugated with Fluorescein (1∶200, Vector Laboratories). For actin visualization we used phalloidin-Fluorescein (2 µM, Sigma-Aldrich) solved in PBS.

#### Cell cultures

Cells were fixed at room temperature for 15 min in 4% paraformaldehyde (Sigma-Aldrich Inc.) in PBS. The coverslips were mounted using Vectashield Hard Set medium with DAPI (Vector Laboratories). The total number of neurites and neurites with growth cones were counted (*n* = 3, 10 fields/experiment) and the percentage of growth cone-positive neurites was calculated. Growth cones from three independent experiments were stained with Gap43 antibodies and the size of 30 growth cones in both injured and uninjured cultures was measured using the outline tool in AxioVision 4.8 software (Zeiss). The neurites were stained with βIII tubulin and the number of branches and the total neurite length of 50 cells in uninjured and injured cultures of three independent experiments was measured using the curve tool in AxioVision 4.8 software (Zeiss). Statistical analysis was performed using two-tailed t test in GraphPad Prism 5 (GraphPad Software, Inc.). To elucidate which cell types were proliferating after injury, the total number of Ki67-positive cells and the number of cells that were double-labeled for Ki67 and either βIII tubulin, GFAP or CNPase were counted (*n* = 3, 10 fields/experiment) and the percentage ± SEM of proliferating cells of each cell type calculated.

#### Brain tissue

Permeabilization and blocking was performed for 1 h in RT in 0.3% Triton X-100 in PBS (0.3% Triton/PBS) and 5% NGS. Primary antibodies were diluted in 0.3% Triton/PSB with 0.5% NGS and incubated O/N in 4°C. Sections were thoroughly washed in 0.3% Triton/PBS and incubated on shaker in RT with secondary antibody for 1 h and washed in 0.3% Triton/PBS. The sections were mounted on glass with Vectashield containing DAPI (Vector).

### Western blotting

Primary antibodies against the following targets were used: Rabbit Ezrin-Moesin-Radixin (ERM, diluted to 1∶1000, Cell Signaling), Rabbit Phosphorylated-Ezrin-Moesin-Radixin (pERM, diluted to 1∶1000, Cell Signaling) rabbit β-actin, rabbit Histone H2B and rabbit GAPDH (0.25 µg/ml, all from Imgenex). Secondary antibodies used: HRP-linked antibody against rabbit (1∶50,000, GE Healthcare).

#### Cell cultures

Cell lysates were made in lysis buffer (20 mM Tris pH 7.5, 0.5% Triton-X-100, 0.5% Deoxycholic acid, 150 mM NaCl, 10 mM EDTA, 30 mM NaPyroP) supplemented with 15% protease inhibitor cocktail (Roche) and sodium orthovanadate (Na_3_VO_4_) (0.1 M, Sigma). Twenty micrograms of total cell lysates from uninjured (*n* = 6) and injured (*n* = 6) cell cultures were prepared and analyzed for actin expression. An average ratio was calculated as a mean of the six independent experiments and given as a mean ratio ± SEM.

#### Brain tissue

Cortices from the injured hemispheres of mice subjected to TBI (*n* = 5) and naive controls (*n* = 5) were homogenized in lysis buffer. From the lysates, 15 µg of protein per sample was analyzed by Western blot. The membrane was developed using enhanced chemiluminescence (ECL) system (GE Healthcare) and the intensity of the bands was analyzed with the gel analysis tool in Image J software. Stripping was performed in sodium hydroxide (0.4 M) for 10 min in room temperature. The ERM expression was normalized against GAPDH expression before calculating a ratio between ERM and pERM. The five independent experiments for naïve respective CCI animals are given as a mean ratio ± SEM. The average increase in pERM after TBI relative pERM in naïve animals was calculated as a ratio between the average expression of the five injured compared to the uninjured (naïve) animals.

### Time-lapse experiments

The time-lapse experiments were carried out using a Nikon Biostation IM Live Cell Recorder apparatus where the cells are kept at 37°C, humidified 5% CO_2_ in air. The cell culture was left unharmed (uninjured controls) or injured by a scalpel cut just before the experiment as described above. The experiments ran for 24 h with pictures taken every 5 min.

### Chemicals and reagents for MS analysis

Acetonitrile (ACN), methanol, acetic acid (HAc), formic acid (FA) and ammonium bicarbonate (NH_4_HCO_3_) were from Merck and protease inhibitor cocktail, N-octyl-β-D-glucopyranoside (β-OG) and trifluoroacetic acid (TFA) were purchased from Sigma Aldrich. For tryptic digestion, iodoacetamide (IAA) and dithiothreitol (DTT) (Sigma Aldrich) and trypsin (sequencing grade from bovine pancreas 1418475; Roche diagnostic) were used. Ultrapure water was prepared by Milli-Q water purification system (Millipore).

### Protein extraction from the collected cell suspensions

Cells were homogenized for 60 seconds in a blender (POLYTRON PT 1200, Kinematica) with 1 mL of N-octyl-β-D-glucopyranoside lysis buffer (1% β-OG, 10 mM Tris-HCl pH 7.4, 0.15 M NaCl, 1 mM EDTA). After homogenization, the samples were incubated for 1 hour at 4°C during mild agitation. The cell lysates were clarified by centrifugation for 30 min (10000× g at 4°C) using a Sigma 2K15 ultracentrifuge (Sigma Laborzentrifugen GmbH). The supernatant was collected and further processed.

### Protein quantification

The total protein content of extracted proteins from cells samples was determined using the DC Protein Assay Kit (BioRad Laboratories), which is based on the modified Lowry method with bovine serum albumin as standard [41]. The DC assay was carried out according to the manufacturer's instructions using 96-well microtiter plate reader model 680 (BioRad Laboratories).

### On-filter digestion of the collected cell suspensions

A normalized protein amount corresponding to 35 µg was processed with a previously described on-filter digestion protocol [42] using 3 kDa filters (Pall Life Sciences). The samples were reduced, alkylated, and on-filter digested as described in [43]. The collected filtrates were vacuum centrifuged to dryness using a Speedvac system ISS110 (Thermo Scientific). Prior to nanoLC-MS/MS analysis, the samples were redissolved in 0.1% TFA to yield an approximate tryptic peptides concentration of 0.3 µg/µL.

### On-filter desalting and digestion of the excreted proteins in the grow media

Aliquots of 500 µL collected cell growth media samples were processed with a previously described on-filter digestion protocol [42] using 3 kDa filters (Pall Life Sciences). The samples were reduced, alkylated, and on-filter digested as described in [43].

### NanoLC-MS/MS for protein identification

The protein identification experiments were performed using a 7 T hybrid LTQ FT mass spectrometer (ThermoFisher Scientific, Bremen, Germany) fitted with a nano-electrospray ionization (ESI) ion source. On-line nanoLC separations were performed using an Agilent 1100 nanoflow system (Agilent Technologies). The peptide separations were performed on in-house packed 15-cm fused silica emitters (75-µm inner diameter, 375-µm outer diameter). The separations were performed at a flow of 200 nL/min with mobile phases A (water with 0.5% acetic acid) and B (89.5% acetonitrile, 10% water, and 0.5% acetic acid). A 100-minutes gradient from 2% B to 50% B followed by a washing step with 98% B for 5 minutes was used. MS analyses were performed using unattended data-dependent acquisition mode, in which the mass spectrometer automatically switches between acquiring a high resolution survey mass spectrum in the FTMS (resolving power 100 000 FWHM) and consecutive low-resolution, collision-induced dissociation fragmentation of up to five of the most abundant ions in the ion trap. Acquired data (.RAW-files) were converted to the .mgf format using an in-house written program (C++) and subjected to protein identification using MASCOT search engine (version 2.2.2, Matrix Science) against the SwissProt database version 51.6. The search parameters were set to Taxonomy: Mus musculus, Enzyme: Trypsin, Fixed modifications: Carbamidomethyl (C), Variable modifications: Oxidation (M) and Deamidated (NQ), Peptide tolerance: 0.03 Da, MS/MS tolerance: 0.8 Da and maximum 2 missed cleavage sites. Proteins were only considered to be positively matched if they passed the stringent MudPIT MASCOT ion scoring (p≤0.05) and at least one peptide passed the required bold red criteria.

### Data analysis

The subcellular location of the identified proteins was elucidated by collection of information from the Uniprot database.

## Supporting Information

Figure S1 **Two independent MS analyses of uninjured and injured medium and cell fractions were run to verify proteins the proteins found.** The subcellular location of the identified proteins was elucidated by collection of information from the Uniprot database and displayed in pie charts. (A) In the medium of uninjured cells we found a total of 165 overlapping proteins and (B) 155 proteins in the injured culture medium. (C) In the cell fractions, 323 proteins were found in the uninjured cells and (D) 275 in the injured cells.
(TIF)

Figure S2 **Neurons and oligodendrocytes appear almost completely devoid of ERM and pERM expression.** Stainings against ERM or pERM together with either the neuronal marker βIII tubulin or the oligodendrocytic marker CNPase reveal little to no overlap of ERM or pERM with neither neurons nor oligodendrocytes.
(TIF)

Figure S3 **ERM and pERM surrounds ingested dead, cells in astrocytes, both in vitro (A) and in vivo (B).**
(DOC)

Table S1 **References to previously shown functions for the proteins found in medium exclusively after injury.**
(DOC)

Table S2 **References to previously shown functions for the proteins found in cells exclusively after injury.**
(DOC)

Video S1 **Injury induces proliferation and migration towards the cut in neuronal cells.** Neurons, recognized by their round to oval somal shape and their extending axon and dendrites, migrate towards and along the cut. They also proliferate at a higher ratio compared to neurons in an uninjured culture. One should note, though, that although expressing the neuronal marker βIII tubulin, the cells are not completely mature. The astrocytes are recognized by their round cell nuclei and often highly vacuolized appearance and are frequently covered in dead cells and debris. They extend numerous lamellipodia towards the laceration, but do not actively migrate towards it. Astrocytes are not induced to proliferate in response to injury in comparison to the neurons. Oligodendrocytes are few and harder to recognize, but are neither induced to migrate nor do they proliferate. Films are composed at 7 frames per second with images taken every 10 minutes for 24 h.
(MP4)

Video S2 **Uninjured cell cultures display less proliferation and no directional migration.** Neurons, recognized by their round to oval somal shape and their extending axon and dendrites, migrate aimlessly and less fervently in uninjured cultures than injured ones. Although, proliferation is observed also in uninjured cultures (likely due to the relative immaturity of the neurons) less cell divisions are observed in uninjured cultures compared to injured ones. The astrocytes are recognized by their round cell nuclei and often highly vacuolized appearance and are frequently covered in dead cells and debris. In uninjured cultures, astrocytes are mostly concerned with clearing up the cellular debris and do not actively migrate or proliferate. No oligodendrocyte migration or proliferation was detected. Films are composed at 7 frames per second with images taken every 10 minutes for 24 h.
(MP4)
