# Supplementary figures and images for: Identification of Injury Specific Proteins in a Cell Culture Model of Traumatic Brain Injury
Source: PLoS One. 2013 Feb 7;8(2):e55983. doi: 10.1371/journal.pone.0055983 (PMC3567017; doi:10.1371/journal.pone.0055983)

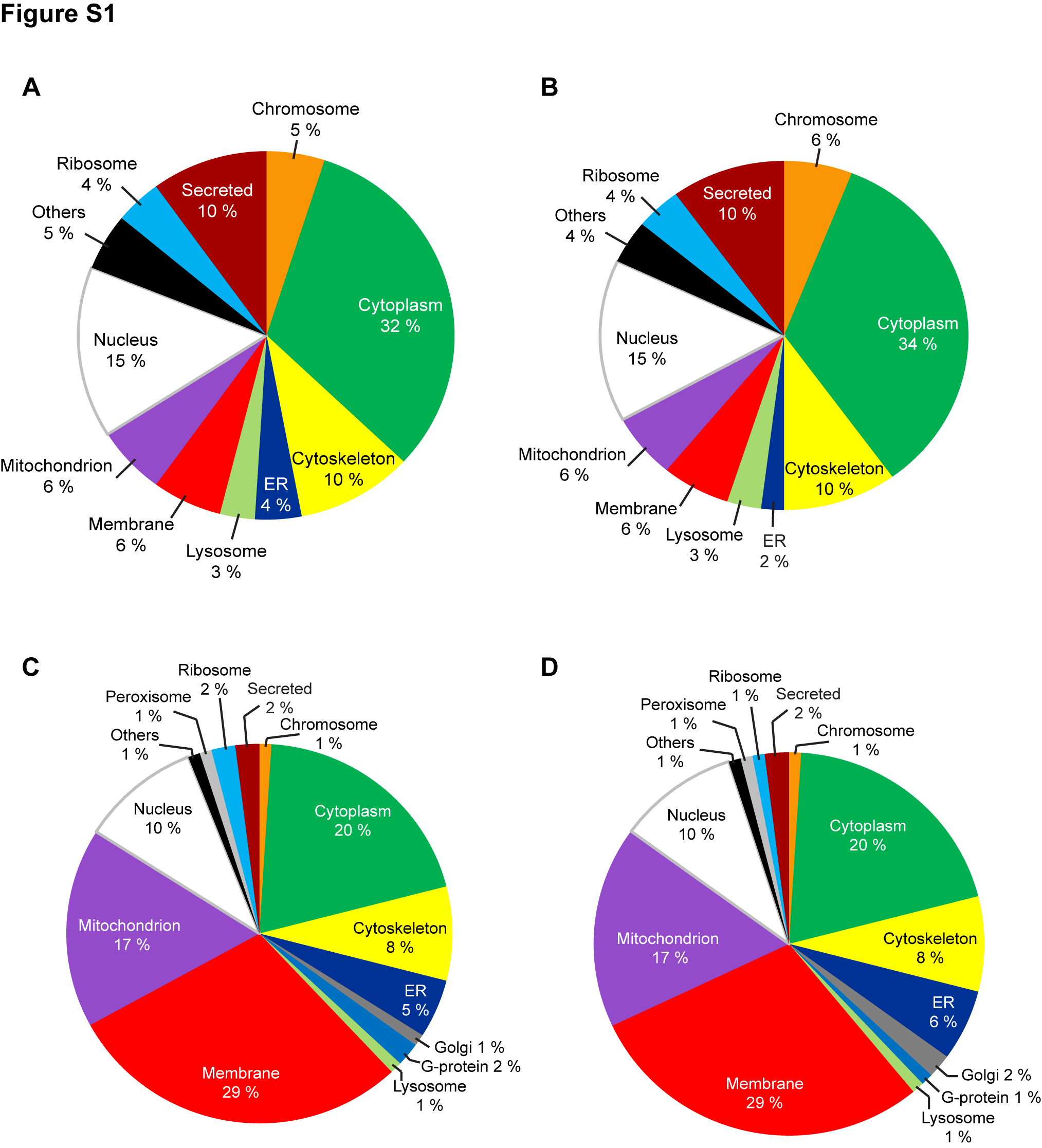

Supplement: Figure S1 — Two independent MS analyses of uninjured and injured medium and cell fractions were run to verify proteins the proteins found. The subcellular location of the identified proteins was elucidated by collection of information from the Uniprot database and displayed in pie charts. (A) In the medium of uninjured cells we found a total of 165 overlapping proteins and (B) 155 proteins in the injured culture medium. (C) In the cell fractions, 323 proteins were found in the uninjured cells and (D) 275 in the injured cells. (TIF) [file pone.0055983.s001.tif]

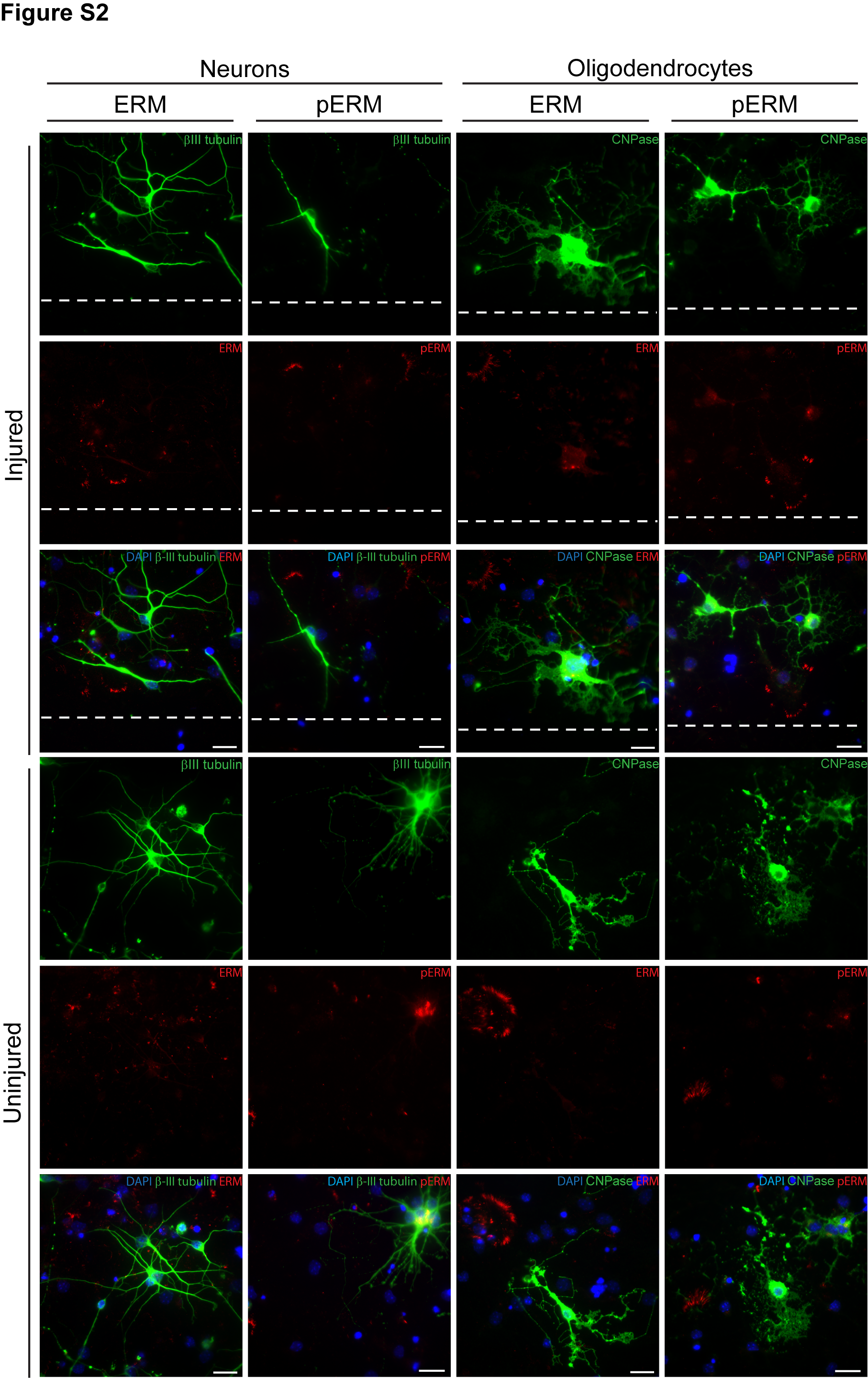

Supplement: Figure S2 — Neurons and oligodendrocytes appear almost completely devoid of ERM and pERM expression. Stainings against ERM or pERM together with either the neuronal marker βIII tubulin or the oligodendrocytic marker CNPase reveal little to no overlap of ERM or pERM with neither neurons nor oligodendrocytes. (TIF) [file pone.0055983.s002.tif]
