# Supplementary material for: Identification of Injury Specific Proteins in a Cell Culture Model of Traumatic Brain Injury
Source: PLoS One. 2013 Feb 7;8(2):e55983. doi: 10.1371/journal.pone.0055983 (PMC3567017; doi:10.1371/journal.pone.0055983)
Supplement: Figure S3 — ERM and pERM surrounds ingested dead, cells in astrocytes, both in vitro (A) and in vivo (B). (DOC) [file pone.0055983.s003.doc]

# SUPPORTING INFORMATION

## Figure S1

Two independent MS analyses of uninjured and injured medium and cell fractions were run to verify proteins the proteins found. The subcellular location of the identified proteins was elucidated by collection of information from the Uniprot database and displayed in pie charts. (A) In the medium of uninjured cells we found a total of 165 overlapping proteins and (B) 155 proteins in the injured culture medium. (C) In the cell fractions, 323 proteins were found in the uninjured cells and (D) 275 in the injured cells.

## Figure S2

Neurons and oligodendrocytes appear almost completely devoid of ERM and pERM expression. Stainings against ERM or pERM together with either the neuronal marker βIII tubulin or the oligodendrocytic marker CNPase reveal little to no overlap of ERM or pERM with neither neurons nor oligodendrocytes.

## Figure S3

ERM and pERM surrounds ingested dead, cells in astrocytes, both *in vitro* (A) and *in vivo* (B).

## Video S1

Injury induces proliferation and migration towards the cut in neuronal cells. Neurons, recognized by their round to oval somal shape and their extending axon and dendrites, migrate towards and along the cut. They also proliferate at a higher ratio compared to neurons in an uninjured culture. One should note, though, that although expressing the neuronal marker βIII tubulin, the cells are not completely mature. The astrocytes are recognized by their round cell nuclei and often highly vacuolized appearance and are frequently covered in dead cells and debris. They extend numerous lamellipodia towards the laceration, but do not actively migrate towards it. Astrocytes are not induced to proliferate in response to injury in comparison to the neurons. Oligodendrocytes are few and harder to recognize, but are neither induced to migrate nor do they proliferate. Films are composed at 7 frames per second with images taken every 10 minutes for 24 h.

## Video S2

Uninjured cell cultures display less proliferation and no directional migration. Neurons, recognized by their round to oval somal shape and their extending axon and dendrites, migrate aimlessly and less fervently in uninjured cultures than injured ones. Although, proliferation is observed also in uninjured cultures (likely due to the relative immaturity of the neurons) less cell divisions are observed in uninjured cultures compared to injured ones. The astrocytes are recognized by their round cell nuclei and often highly vacuolized appearance and are frequently covered in dead cells and debris. In uninjured cultures, astrocytes are mostly concerned with clearing up the cellular debris and do not actively migrate or proliferate. No oligodendrocyte migration or proliferation was detected. Films are composed at 7 frames per second with images taken every 10 minutes for 24 h.
