# Supplementary material for: Identification of Injury Specific Proteins in a Cell Culture Model of Traumatic Brain Injury
Source: PLoS One. 2013 Feb 7;8(2):e55983. doi: 10.1371/journal.pone.0055983 (PMC3567017; doi:10.1371/journal.pone.0055983)
Supplement: Table S1 — References to previously shown functions for the proteins found in medium exclusively after injury. (DOC) [file pone.0055983.s004.doc]

**Table S1. References to previously shown functions** for the proteins found in medium exclusively after injury.

| **Accession (_MOUSE)** | **1433G** | **H12** | **ALDOA** | **GDIA** | **NDKA** | **EZRI** | **FAS** | **LGMN** | **PRDX1** | **LXN** | **LDHA** |
| --- | --- | --- | --- | --- | --- | --- | --- | --- | --- | --- | --- |
| **Protein description** | **14-3-3 protein gamma** | **Histone H1.2** | **Fructose-bisphosphate aldolase A** | **Rab GDP dissociation inhibitor alpha** | **Nucleoside diphosphate kinase A** | **Ezrin** | **Fatty acid synthase** | **Legumain** | **Peroxiredoxin-1** | **Latexin** | **L-lactate dehydro-genase A chain** |
| **Actin** |  |  | [1] |  |  | [2,3,4] |  |  |  |  |  |
| **Neurological disease/ degeneration/ TBI** | [5,6,7,8,9] |  | [10,11] | [12,13] | [14,15] | [4,16] | [17,18] |  | [19,20,21] | [22,23,24] |  |
| **Scar formation/ Reactive gliosis** | [7] |  |  |  |  | [2] |  |  |  |  |  |
| **Migration/ Motility/ Chemotaxis** |  |  |  |  | [25] | [4] |  | [26] |  |  | [27] |
| **Proliferation/ Differentiation/ Cell death/ Survival** | [6] | [28,29,30,31,32,33] | [34,35,36] |  | [37] | [38] |  |  |  | [22,39,40] |  |
| **Engulfment/ Degradation** |  |  |  |  |  | [3,41,42] | [43] |  |  |  |  |
| **Neurite/ Growth cones** |  |  | [44] |  | [25,37] |  |  |  |  |  |  |
| **ER/Golgi/ Secretion/ Energy metabolism** | [45] |  |  | [13,46,47] |  |  | [48] | [26] | [21,49,50] |  |  |
| **Immune response** |  |  | [10] |  |  | [2,4] |  | [26,51,52] | [21,50,53] |  |  |

| **Accession (_MOUSE)** | **MOES** | **COF1** | **FHL1** | **H11** | **NACAM** | **IDHC** | **GDIR1** | **ASGL1** | **PARK7** | **CNN3** | **6PGD** |
| --- | --- | --- | --- | --- | --- | --- | --- | --- | --- | --- | --- |
| **Protein description** | **Moesin** | **Cofilin-1** | **Four and a half LIM domains protein 1** | **Histone H1.1** | **Nascent polypeptide-associated complex subunit alpha, muscle-specific form** | **Isocitrate dehydrogenase [NADP] cytoplasmic** | **Rho GDP-dissociation inhibitor 1** | **L-aspara-ginase** | **Protein DJ-1** | **Calponin-3** | **6-phospho-gluconate dehydrogenase, decarboxylating** |
| **Actin** | [1,2] | [3,4,5,6,7] | [8] |  |  |  | [9] |  |  | [10,11,12,13] |  |
| **Neurological disease/ degeneration/ TBI** | [14] | [3,4,7,15] |  |  | [16] |  | [17] |  | [18,19,20] | [11,13,21] | [22] |
| **Scar formation/ Reactive gliosis** | [23] |  |  |  |  |  |  |  |  | [10] |  |
| **Migration/ Motility/ Chemotaxis** | [1,2] |  |  |  |  |  | [9,24,25] |  |  | [12,13] |  |
| **Proliferation/ Differentiation/ Cell death/Survival** | [1] |  |  | [26] | [16,27,28,29,30,31] |  | [9,24,32,33] |  | [19,20] | [13,21] |  |
| **Engulfment/ Degradation** | [1,34] |  |  |  |  |  |  |  |  |  |  |
| **Neurite/ Growth cones** |  |  |  |  |  |  |  |  |  | [13] |  |
| **ER/Golgi/ Secretion/ Energy metabolism** |  |  |  |  | [16] | [35] |  |  | [20,36] |  | [22] |
| **Immune response** | [14,23] | [5] |  |  | [29,31] |  |  |  | [19] |  |  |

| **Accession (_MOUSE)** | **FTHFD** | **DDAH1** | **DDX17** | **NDRG2** | **ARP2** | **LAMP1** | **2AAA** | **PSA5** | **HINT1** |
| --- | --- | --- | --- | --- | --- | --- | --- | --- | --- |
| **Protein description** | **10-formyltetra-hydrofolate dehydro-genase** | **N(G),N(G)-dimethyl-arginine dimethylamino-hydrolase 1** | **Probable ATP-dependent RNA helicase DDX17** | **Protein NDRG2** | **Actin-related protein 2** | **Lysosome-associated membrane glycoprotein 1** | **Serine/threonine-protein phosphatase 2A 65 kDa regulatory subunit A alpha isoform** | **Proteasome subunit alpha type-5** | **Histidine triad nucleotide-binding protein 1** |
| **Actin** |  | [37,38] |  | [39] | [40,41,42] |  |  |  |  |
| **Neurological disease/ degeneration/ TBI** | [43] |  |  | [39,44,45,46] |  |  |  | [47] | [48] |
| **Scar formation/ Reactive gliosis** |  |  |  | [39] |  |  |  |  |  |
| **Migration/ Motility/ Chemotaxis** |  | [38,49] |  |  | [40] |  |  |  |  |
| **Proliferation/ Differentiation/ Cell death/Survival** | [50,51] | [38,52] | [53,54] | [39,44,45] | [42] | [55] |  | [47] |  |
| **Engulfment/ Degradation** |  |  |  |  | [40] | [56] |  |  |  |
| **Neurite/ Growth cones** |  |  |  | [39,57] | [42] |  |  |  |  |
| **ER/Golgi/ Secretion/ Energy metabolism** |  | [52] |  |  |  |  |  |  |  |
| **Immune response** |  |  |  |  |  | [55] |  |  |  |

| **Accession (_MOUSE)** | **FKB1A** | **MTPN** | **SUMO2** | **TAU** | **RL7** | **MARCS** | **FBX2** | **IBP2** | **GLNA** | **TYB4** |
| --- | --- | --- | --- | --- | --- | --- | --- | --- | --- | --- |
| **Protein description** | **Peptidyl-prolyl cis-trans isomerase FKBP1A** | **Myotrophin** | **Small ubiquitin-related modifier 2** | **Microtubule-associated protein tau** | **60S ribosomal protein L7** | **Myristoylated alanine-rich C-kinase substrate** | **F-box only protein 2** | **Insulin-like growth factor-binding protein 2** | **Glutamine synthetase** | **Thymosin beta-4** |
| **Actin** |  | [41] |  |  |  | [58,59,60] |  |  |  | [61] |
| **Neurological disease/ degeneration/ TBI** | [62] |  | [63,64] | [65,66,67] |  | [60,68] | [69,70] | [71,72] | [73,74] | [75] |
| **Scar formation/ Reactive gliosis** |  |  |  |  |  |  |  | [71,76] | [10,77] |  |
| **Migration/ Motility/ Chemotaxis** |  |  |  |  |  | [58,78] |  |  | [74] | [79] |
| **Proliferation/ Differentiation/ Cell death/Survival** | [62] | [80] | [64] |  |  | [78] | [81] | [72,76,82] | [10] | [75,79] |
| **Engulfment/ Degradation** |  |  |  |  |  | [58] | [81] |  |  |  |
| **Neurite/ Growth cones** |  |  |  |  |  | [59] |  |  |  |  |
| **ER/Golgi/ Secretion/ Energy metabolism** | [62] |  |  |  |  | [58] |  | [72] | [73,83] | [79,84] |
| **Immune response** | [62] |  |  |  |  | [68] |  |  |  | [61,79] |

| **Accession (_MOUSE)** | **NP1L4** | **RS11** | **HNRDL** | **RL5** | **IF2G** | **DEST** | **AP2B1** | **FPPS** | **WDR1** | **RL17** | **FSTL1** | **TCPG** |
| --- | --- | --- | --- | --- | --- | --- | --- | --- | --- | --- | --- | --- |
| **Protein description** | **Nucleosome assembly protein 1-like 4** | **40S ribo-somal protein S11** | **Hetero-geneous nuclear ribonucleo-protein D-like** | **60S ribo-somal protein L5** | **Eukaryotic translation initiation factor 2 subunit 3, X-linked** | **Destrin** | **AP-2 complex subunit beta** | **Farnesyl pyrophos-phate synthase** | **WD repeat-containing protein 1** | **60S ribosomal protein L17** | **Follistatin-related protein 1** | **T-complex protein 1 subunit gamma** |
| **Actin** |  |  |  |  |  | [85,86] |  |  | [87,88] |  |  |  |
| **Neurological disease/ degeneration/ TBI** |  |  |  | [89] |  | [85] |  |  |  |  | [90,91] | [92] |
| **Scar formation/ Reactive gliosis** |  |  |  |  |  |  |  |  |  |  |  |  |
| **Migration/ Motility/ Chemotaxis** |  |  |  |  |  | [85] |  |  | [88] |  | [93] |  |
| **Proliferation/ Differentiation/ Cell death/ Survival** |  |  |  |  |  | [86] |  | [94] | [87,88] |  | [93,95] |  |
| **Engulfment/ Degradation** |  |  |  |  |  | [85] |  |  | [87] |  |  |  |
| **Neurite/ Growth cones** |  |  |  |  |  | [85] |  |  |  |  |  |  |
| **ER/Golgi/ Secretion/ Energy metabolism** |  |  |  |  |  |  |  |  |  |  | [90] |  |
| **Immune response** |  |  |  |  |  |  |  |  |  |  |  |  |

1. Niggli V, Rossy J (2008) Ezrin/radixin/moesin: versatile controllers of signaling molecules and of the cortical cytoskeleton. Int J Biochem Cell Biol 40: 344-349.

2. Persson A, Lindwall C, Curtis MA, Kuhn HG (2010) Expression of ezrin radixin moesin proteins in the adult subventricular zone and the rostral migratory stream. Neuroscience 167: 312-322.

3. Whiteman IT, Gervasio OL, Cullen KM, Guillemin GJ, Jeong EV, et al. (2009) Activated actin-depolymerizing factor/cofilin sequesters phosphorylated microtubule-associated protein during the assembly of alzheimer-like neuritic cytoskeletal striations. J Neurosci 29: 12994-13005.

4. Bamburg JR, Bernstein BW, Davis RC, Flynn KC, Goldsbury C, et al. (2010) ADF/Cofilin-actin rods in neurodegenerative diseases. Curr Alzheimer Res 7: 241-250.

5. Dwivedi S, Pandey D, Khandoga AL, Brandl R, Siess W (2010) Rac1-mediated signaling plays a central role in secretion-dependent platelet aggregation in human blood stimulated by atherosclerotic plaque. J Transl Med 8: 128.

6. Popova EN, Pletjushkina OY, Dugina VB, Domnina LV, Ivanova OY, et al. (2010) Scavenging of reactive oxygen species in mitochondria induces myofibroblast differentiation. Antioxid Redox Signal 13: 1297-1307.

7. Campbell JN, Low B, Kurz JE, Patel SS, Young MT, et al. (2011) Mechanisms of Dendritic Spine Remodeling in a Rat Model of Traumatic Brain Injury. J Neurotrauma.

8. Shathasivam T, Kislinger T, Gramolini AO (2010) Genes, proteins and complexes: the multifaceted nature of FHL family proteins in diverse tissues. J Cell Mol Med 14: 2702-2720.

9. Ishizaki H, Togawa A, Tanaka-Okamoto M, Hori K, Nishimura M, et al. (2006) Defective chemokine-directed lymphocyte migration and development in the absence of Rho guanosine diphosphate-dissociation inhibitors alpha and beta. J Immunol 177: 8512-8521.

10. Egnaczyk GF, Pomonis JD, Schmidt JA, Rogers SD, Peters C, et al. (2003) Proteomic analysis of the reactive phenotype of astrocytes following endothelin-1 exposure. Proteomics 3: 689-698.

11. Kreipke CW, Morgan NC, Petrov T, Rafols JA (2006) Calponin and caldesmon cellular domains in reacting microvessels following traumatic brain injury. Microvasc Res 71: 197-204.

12. Wu KC, Jin JP (2008) Calponin in non-muscle cells. Cell Biochem Biophys 52: 139-148.

13. Kreipke CW, Rafols JA (2009) Calponin control of cerebrovascular reactivity: therapeutic implications in brain trauma. J Cell Mol Med 13: 262-269.

14. Moon Y, Kim JY, Choi SY, Kim K, Kim H, et al. (2011) Induction of ezrin-radixin-moesin molecules after cryogenic traumatic brain injury of the mouse cortex. Neuroreport 22: 304-308.

15. Yao J, Hennessey T, Flynt A, Lai E, Beal MF, et al. (2010) MicroRNA-related cofilin abnormality in Alzheimer's disease. PLoS One 5: e15546.

16. Hotokezaka Y, van Leyen K, Lo EH, Beatrix B, Katayama I, et al. (2009) alphaNAC depletion as an initiator of ER stress-induced apoptosis in hypoxia. Cell Death Differ 16: 1505-1514.

17. Weitzdoerfer R, Stolzlechner D, Dierssen M, Ferreres J, Fountoulakis M, et al. (2001) Reduction of nucleoside diphosphate kinase B, Rab GDP-dissociation inhibitor beta and histidine triad nucleotide-binding protein in fetal Down syndrome brain. J Neural Transm Suppl: 347-359.

18. Allard L, Burkhard PR, Lescuyer P, Burgess JA, Walter N, et al. (2005) PARK7 and nucleoside diphosphate kinase A as plasma markers for the early diagnosis of stroke. Clin Chem 51: 2043-2051.

19. Waak J, Weber SS, Waldenmaier A, Gorner K, Alunni-Fabbroni M, et al. (2009) Regulation of astrocyte inflammatory responses by the Parkinson's disease-associated gene DJ-1. FASEB J 23: 2478-2489.

20. Mullett SJ, Hinkle DA (2011) DJ-1 deficiency in astrocytes selectively enhances mitochondrial Complex I inhibitor-induced neurotoxicity. J Neurochem 117: 375-387.

21. Natale JE, Ahmed F, Cernak I, Stoica B, Faden AI (2003) Gene expression profile changes are commonly modulated across models and species after traumatic brain injury. J Neurotrauma 20: 907-927.

22. Palmer AM (1999) The activity of the pentose phosphate pathway is increased in response to oxidative stress in Alzheimer's disease. J Neural Transm 106: 317-328.

23. John GR, Chen L, Rivieccio MA, Melendez-Vasquez CV, Hartley A, et al. (2004) Interleukin-1beta induces a reactive astroglial phenotype via deactivation of the Rho GTPase-Rock axis. J Neurosci 24: 2837-2845.

24. Qi YX, Qu MJ, Long DK, Liu B, Yao QP, et al. (2008) Rho-GDP dissociation inhibitor alpha downregulated by low shear stress promotes vascular smooth muscle cell migration and apoptosis: a proteomic analysis. Cardiovasc Res 80: 114-122.

25. Chianale F, Rainero E, Cianflone C, Bettio V, Pighini A, et al. (2010) Diacylglycerol kinase alpha mediates HGF-induced Rac activation and membrane ruffling by regulating atypical PKC and RhoGDI. Proc Natl Acad Sci U S A 107: 4182-4187.

26. Terme JM, Sese B, Millan-Arino L, Mayor R, Belmonte JC, et al. (2011) Histone H1 variants are differentially expressed and incorporated into chromatin during differentiation and reprogramming to pluripotency. J Biol Chem 286: 35347-35357.

27. Yotov WV, St-Arnaud R (1996) Differential splicing-in of a proline-rich exon converts alphaNAC into a muscle-specific transcription factor. Genes Dev 10: 1763-1772.

28. Stilo R, Liguoro D, di Jeso B, Leonardi A, Vito P (2003) The alpha-chain of the nascent polypeptide-associated complex binds to and regulates FADD function. Biochem Biophys Res Commun 303: 1034-1041.

29. Al-Shanti N, Aldahoodi Z (2006) Inhibition of alpha nascent polypeptide associated complex protein may induce proliferation, differentiation and enhance the cytotoxic activity of human CD8+ T cells. J Clin Immunol 26: 457-464.

30. Park CY, Pierce SA, von Drehle M, Ivey KN, Morgan JA, et al. (2010) skNAC, a Smyd1-interacting transcription factor, is involved in cardiac development and skeletal muscle growth and regeneration. Proc Natl Acad Sci U S A 107: 20750-20755.

31. Heratizadeh A, Mittermann I, Balaji H, Wichmann K, Niebuhr M, et al. (2011) The role of T-cell reactivity towards the autoantigen alpha-NAC in atopic dermatitis. Br J Dermatol 164: 316-324.

32. Wei L, Imanaka-Yoshida K, Wang L, Zhan S, Schneider MD, et al. (2002) Inhibition of Rho family GTPases by Rho GDP dissociation inhibitor disrupts cardiac morphogenesis and inhibits cardiomyocyte proliferation. Development 129: 1705-1714.

33. Yamashita T, Tohyama M (2003) The p75 receptor acts as a displacement factor that releases Rho from Rho-GDI. Nat Neurosci 6: 461-467.

34. Erwig LP, McPhilips KA, Wynes MW, Ivetic A, Ridley AJ, et al. (2006) Differential regulation of phagosome maturation in macrophages and dendritic cells mediated by Rho GTPases and ezrin-radixin-moesin (ERM) proteins. Proc Natl Acad Sci U S A 103: 12825-12830.

35. Dudley RW, Khairallah M, Mohammed S, Lands L, Des Rosiers C, et al. (2006) Dynamic responses of the glutathione system to acute oxidative stress in dystrophic mouse (mdx) muscles. Am J Physiol Regul Integr Comp Physiol 291: R704-710.

36. Pardo M, Garcia A, Thomas B, Pineiro A, Akoulitchev A, et al. (2006) The characterization of the invasion phenotype of uveal melanoma tumour cells shows the presence of MUC18 and HMG-1 metastasis markers and leads to the identification of DJ-1 as a potential serum biomarker. Int J Cancer 119: 1014-1022.

37. Wojciak-Stothard B, Torondel B, Zhao L, Renne T, Leiper JM (2009) Modulation of Rac1 activity by ADMA/DDAH regulates pulmonary endothelial barrier function. Mol Biol Cell 20: 33-42.

38. Fiedler LR, Wojciak-Stothard B (2009) The DDAH/ADMA pathway in the control of endothelial cell migration and angiogenesis. Biochem Soc Trans 37: 1243-1247.

39. Takeichi T, Takarada-Iemata M, Hashida K, Sudo H, Okuda T, et al. (2011) The effect of Ndrg2 expression on astroglial activation. Neurochem Int 59: 21-27.

40. Insall R, Muller-Taubenberger A, Machesky L, Kohler J, Simmeth E, et al. (2001) Dynamics of the Dictyostelium Arp2/3 complex in endocytosis, cytokinesis, and chemotaxis. Cell Motil Cytoskeleton 50: 115-128.

41. Cooper JA, Sept D (2008) New insights into mechanism and regulation of actin capping protein. Int Rev Cell Mol Biol 267: 183-206.

42. Korobova F, Svitkina T (2008) Arp2/3 complex is important for filopodia formation, growth cone motility, and neuritogenesis in neuronal cells. Mol Biol Cell 19: 1561-1574.

43. Cains S, Shepherd A, Nabiuni M, Owen-Lynch PJ, Miyan J (2009) Addressing a folate imbalance in fetal cerebrospinal fluid can decrease the incidence of congenital hydrocephalus. J Neuropathol Exp Neurol 68: 404-416.

44. Nichols NR (2003) Ndrg2, a novel gene regulated by adrenal steroids and antidepressants, is highly expressed in astrocytes. Ann N Y Acad Sci 1007: 349-356.

45. Li Y, Shen L, Cai L, Wang Q, Hou W, et al. (2011) Spatial-temporal expression of NDRG2 in rat brain after focal cerebral ischemia and reperfusion. Brain Res 1382: 252-258.

46. Li L, Wang J, Shen X, Wang L, Li X, et al. (2011) Expression and prognostic value of NDRG2 in human astrocytomas. J Neurol Sci 308: 77-82.

47. Bedford L, Hay D, Devoy A, Paine S, Powe DG, et al. (2008) Depletion of 26S proteasomes in mouse brain neurons causes neurodegeneration and Lewy-like inclusions resembling human pale bodies. J Neurosci 28: 8189-8198.

48. Varadarajulu J, Schmitt A, Falkai P, Alsaif M, Turck CW, et al. (2011) Differential expression of HINT1 in schizophrenia brain tissue. Eur Arch Psychiatry Clin Neurosci.

49. Wojciak-Stothard B, Torondel B, Tsang LY, Fleming I, Fisslthaler B, et al. (2007) The ADMA/DDAH pathway is a critical regulator of endothelial cell motility. J Cell Sci 120: 929-942.

50. Martinasevic MK, Rios GR, Miller MW, Tephly TR (1999) Folate and folate-dependent enzymes associated with rat CNS development. Dev Neurosci 21: 29-35.

51. Oleinik NV, Krupenko NI, Priest DG, Krupenko SA (2005) Cancer cells activate p53 in response to 10-formyltetrahydrofolate dehydrogenase expression. Biochem J 391: 503-511.

52. Muller WE, Ushijima H, Batel R, Krasko A, Borejko A, et al. (2006) Novel mechanism for the radiation-induced bystander effect: nitric oxide and ethylene determine the response in sponge cells. Mutat Res 597: 62-72.

53. Kern TS, Engerman RL (1991) Retinal polyol and myo-inositol in galactosemic dogs given an aldose-reductase inhibitor. Invest Ophthalmol Vis Sci 32: 3175-3177.

54. Wortham NC, Ahamed E, Nicol SM, Thomas RS, Periyasamy M, et al. (2009) The DEAD-box protein p72 regulates ERalpha-/oestrogen-dependent transcription and cell growth, and is associated with improved survival in ERalpha-positive breast cancer. Oncogene 28: 4053-4064.

55. Kannan K, Stewart RM, Bounds W, Carlsson SR, Fukuda M, et al. (1996) Lysosome-associated membrane proteins h-LAMP1 (CD107a) and h-LAMP2 (CD107b) are activation-dependent cell surface glycoproteins in human peripheral blood mononuclear cells which mediate cell adhesion to vascular endothelium. Cell Immunol 171: 10-19.

56. Huynh KK, Eskelinen EL, Scott CC, Malevanets A, Saftig P, et al. (2007) LAMP proteins are required for fusion of lysosomes with phagosomes. EMBO J 26: 313-324.

57. Takahashi K, Yamada M, Ohata H, Honda K (2005) Ndrg2 promotes neurite outgrowth of NGF-differentiated PC12 cells. Neurosci Lett 388: 157-162.

58. Sundaram M, Cook HW, Byers DM (2004) The MARCKS family of phospholipid binding proteins: regulation of phospholipase D and other cellular components. Biochem Cell Biol 82: 191-200.

59. Yamaguchi H, Shiraishi M, Fukami K, Tanabe A, Ikeda-Matsuo Y, et al. (2009) MARCKS regulates lamellipodia formation induced by IGF-I via association with PIP2 and beta-actin at membrane microdomains. J Cell Physiol 220: 748-755.

60. Su R, Han ZY, Fan JP, Zhang YL (2010) A possible role of myristoylated alanine-rich C kinase substrate in endocytic pathway of Alzheimer's disease. Neurosci Bull 26: 338-344.

61. Paulussen M, Landuyt B, Schoofs L, Luyten W, Arckens L (2009) Thymosin beta 4 mRNA and peptide expression in phagocytic cells of different mouse tissues. Peptides 30: 1822-1832.

62. Kang CB, Hong Y, Dhe-Paganon S, Yoon HS (2008) FKBP family proteins: immunophilins with versatile biological functions. Neurosignals 16: 318-325.

63. Loftus LT, Gala R, Yang T, Jessick VJ, Ashley MD, et al. (2009) Sumo-2/3-ylation following in vitro modeled ischemia is reduced in delayed ischemic tolerance. Brain Res 1272: 71-80.

64. Datwyler AL, Lattig-Tunnemann G, Yang W, Paschen W, Lee SL, et al. (2011) SUMO2/3 conjugation is an endogenous neuroprotective mechanism. J Cereb Blood Flow Metab 31: 2152-2159.

65. McKee AC, Cantu RC, Nowinski CJ, Hedley-Whyte ET, Gavett BE, et al. (2009) Chronic traumatic encephalopathy in athletes: progressive tauopathy after repetitive head injury. J Neuropathol Exp Neurol 68: 709-735.

66. Leoni V, Solomon A, Kivipelto M (2010) Links between ApoE, brain cholesterol metabolism, tau and amyloid beta-peptide in patients with cognitive impairment. Biochem Soc Trans 38: 1021-1025.

67. Tran HT, Sanchez L, Esparza TJ, Brody DL (2011) Distinct temporal and anatomical distributions of amyloid-beta and tau abnormalities following controlled cortical impact in transgenic mice. PLoS One 6: e25475.

68. Hasegawa H, Nakai M, Tanimukai S, Taniguchi T, Terashima A, et al. (2001) Microglial signaling by amyloid beta protein through mitogen-activated protein kinase mediating phosphorylation of MARCKS. Neuroreport 12: 2567-2571.

69. Nelson RF, Glenn KA, Zhang Y, Wen H, Knutson T, et al. (2007) Selective cochlear degeneration in mice lacking the F-box protein, Fbx2, a glycoprotein-specific ubiquitin ligase subunit. J Neurosci 27: 5163-5171.

70. Gong B, Chen F, Pan Y, Arrieta-Cruz I, Yoshida Y, et al. (2010) SCFFbx2-E3-ligase-mediated degradation of BACE1 attenuates Alzheimer's disease amyloidosis and improves synaptic function. Aging Cell 9: 1018-1031.

71. O'Donnell SL, Frederick TJ, Krady JK, Vannucci SJ, Wood TL (2002) IGF-I and microglia/macrophage proliferation in the ischemic mouse brain. Glia 39: 85-97.

72. Chesik D, De Keyser J, Wilczak N (2007) Insulin-like growth factor binding protein-2 as a regulator of IGF actions in CNS: implications in multiple sclerosis. Cytokine Growth Factor Rev 18: 267-278.

73. Albrecht J, Zielinska M, Norenberg MD (2010) Glutamine as a mediator of ammonia neurotoxicity: A critical appraisal. Biochem Pharmacol 80: 1303-1308.

74. Zou J, Wang YX, Mu HJ, Xiang J, Wu W, et al. (2011) Down-regulation of glutamine synthetase enhances migration of rat astrocytes after in vitro injury. Neurochem Int 58: 404-413.

75. Xiong Y, Mahmood A, Meng Y, Zhang Y, Zhang ZG, et al. (2011) Treatment of traumatic brain injury with thymosin beta in rats. J Neurosurg 114: 102-115.

76. Chesik D, Kuhl NM, Wilczak N, De Keyser J (2004) Enhanced production and proteolytic degradation of insulin-like growth factor binding protein-2 in proliferating rat astrocytes. J Neurosci Res 77: 354-362.

77. Goursaud S, Kozlova EN, Maloteaux JM, Hermans E (2009) Cultured astrocytes derived from corpus callosum or cortical grey matter show distinct glutamate handling properties. J Neurochem 108: 1442-1452.

78. Weimer JM, Yokota Y, Stanco A, Stumpo DJ, Blackshear PJ, et al. (2009) MARCKS modulates radial progenitor placement, proliferation and organization in the developing cerebral cortex. Development 136: 2965-2975.

79. Huff T, Muller CS, Otto AM, Netzker R, Hannappel E (2001) beta-Thymosins, small acidic peptides with multiple functions. Int J Biochem Cell Biol 33: 205-220.

80. Das B, Gupta S, Vasanji A, Xu Z, Misra S, et al. (2008) Nuclear co-translocation of myotrophin and p65 stimulates myocyte growth. Regulation by myotrophin hairpin loops. J Biol Chem 283: 27947-27956.

81. Ho MS, Tsai PI, Chien CT (2006) F-box proteins: the key to protein degradation. J Biomed Sci 13: 181-191.

82. Chesik D, De Keyser J, Glazenburg L, Wilczak N (2006) Insulin-like growth factor binding proteins: regulation in chronic active plaques in multiple sclerosis and functional analysis of glial cells. Eur J Neurosci 24: 1645-1652.

83. Lu SC (2009) Regulation of glutathione synthesis. Mol Aspects Med 30: 42-59.

84. Kannan L, Rath NC, Liyanage R, Lay JO, Jr. (2010) Effect of toll-like receptor activation on thymosin beta-4 production by chicken macrophages. Mol Cell Biochem 344: 55-63.

85. Bamburg JR (1999) Proteins of the ADF/cofilin family: essential regulators of actin dynamics. Annu Rev Cell Dev Biol 15: 185-230.

86. Bernstein BW, Bamburg JR (2010) ADF/cofilin: a functional node in cell biology. Trends Cell Biol 20: 187-195.

87. Konzok A, Weber I, Simmeth E, Hacker U, Maniak M, et al. (1999) DAip1, a Dictyostelium homologue of the yeast actin-interacting protein 1, is involved in endocytosis, cytokinesis, and motility. J Cell Biol 146: 453-464.

88. Kato A, Kurita S, Hayashi A, Kaji N, Ohashi K, et al. (2008) Critical roles of actin-interacting protein 1 in cytokinesis and chemotactic migration of mammalian cells. Biochem J 414: 261-270.

89. Huang H, Colella S, Kurrer M, Yonekawa Y, Kleihues P, et al. (2000) Gene expression profiling of low-grade diffuse astrocytomas by cDNA arrays. Cancer Res 60: 6868-6874.

90. Zhang G, Ohsawa Y, Kametaka S, Shibata M, Waguri S, et al. (2003) Regulation of FLRG expression in rat primary astroglial cells and injured brain tissue by transforming growth factor-beta 1 (TGF-beta 1). J Neurosci Res 72: 33-45.

91. Li KC, Wang F, Zhong YQ, Lu YJ, Wang Q, et al. (2011) Reduction of follistatin-like 1 in primary afferent neurons contributes to neuropathic pain hypersensitivity. Cell Res 21: 697-699.

92. Giorgi A, Di Francesco L, Principe S, Mignogna G, Sennels L, et al. (2009) Proteomic profiling of PrP27-30-enriched preparations extracted from the brain of hamsters with experimental scrapie. Proteomics 9: 3802-3814.

93. Liu S, Wang L, Wang W, Lin J, Han J, et al. (2006) TSC-36/FRP inhibits vascular smooth muscle cell proliferation and migration. Exp Mol Pathol 80: 132-140.

94. Woo IS, Eun SY, Kim HJ, Kang ES, Lee JH, et al. (2010) Farnesyl diphosphate synthase attenuates paclitaxel-induced apoptotic cell death in human glioblastoma U87MG cells. Neurosci Lett 474: 115-120.

95. Geng Y, Dong Y, Yu M, Zhang L, Yan X, et al. (2011) Follistatin-like 1 (Fstl1) is a bone morphogenetic protein (BMP) 4 signaling antagonist in controlling mouse lung development. Proc Natl Acad Sci U S A 108: 7058-7063.
