# Supplementary material for: Identification of Injury Specific Proteins in a Cell Culture Model of Traumatic Brain Injury
Source: PLoS One. 2013 Feb 7;8(2):e55983. doi: 10.1371/journal.pone.0055983 (PMC3567017; doi:10.1371/journal.pone.0055983)
Supplement: Table S2 — References to previously shown functions for the proteins found in cells exclusively after injury. (DOC) [file pone.0055983.s005.doc]

**Table S2. References to previously shown functions for the proteins found in cells exclusively after injury**.

| **Accession (_MOUSE)** | **TBB2B** | **HSP72** | **UGGG1** | **DPYL1** | **AT8A1** | **SPCS2** | **GLU2B** | **FTHFD** | **VPP1** | **SC6A1** |
| --- | --- | --- | --- | --- | --- | --- | --- | --- | --- | --- |
| **Protein description** | **Tubulin beta-2B chain** | **Heat shock-related 70 kDa protein 2** | **UDP-glucose:glyco-protein glucosyl-transferase 1** | **Dihydro-pyrimidinase-related protein 1** | **Probable phospholipid-transporting ATPase IA** | **Signal peptidase complex subunit 2** | **Glucosidase 2 subunit beta** | **10-formyl-tetrahydro-folate dehydrogenase** | **V-type proton ATPase 116 kDa subunit a isoform 1** | **Sodium- and chloride-dependent GABA transporter 1** |
| **Actin** |  |  |  |  |  |  |  |  |  |  |
| **Neurological disease/ degeneration/ TBI** | [1,2] | [3] |  | [4,5] |  |  |  | [6] |  | [7,8,9] |
| **Scar formation/ Reactive gliosis** |  |  |  |  |  |  |  |  |  | [8] |
| **Migration/ Motility/ Chemotaxis** | [1,10] |  |  | [11] |  |  |  |  |  |  |
| **Proliferation/ Differentiation/ Cell death/ Survival** | [2,10] | [3,12,13] |  | [4,5] |  |  |  | [14,15] |  |  |
| **Engulfment/ Degradation** |  |  |  |  |  |  |  |  | [16,17] |  |
| **Neurite/ Growth cones** | [2] |  |  | [4,11,18] |  |  |  |  | [19] |  |
| **ER/Golgi/ Secretion/ Energy metabolism** |  | [20] | [21] | [22] |  |  | [23] |  | [19,24,25] | [26] |
| **Immune response** |  | [13] | [27] |  |  |  |  |  | [16] | [28] |

| **Accession (_MOUSE)** | **D3D2** | **MYPR** | **CNTN1** | **LDHA** | **ADDG** | **ALDOA** | **RUVB2** | **DHB12** | **ERMP1** | **RL19** |
| --- | --- | --- | --- | --- | --- | --- | --- | --- | --- | --- |
| **Protein description** | **3,2-trans-enoyl-CoA isomerase, mitochondrial** | **Myelin proteolipid protein** | **Contactin-1** | **L-lactate dehydro-genase A chain** | **Gamma-adducin** | **Fructose-bisphosphate aldolase A** | **RuvB-like 2** | **Estradiol 17-beta-dehydro-genase 12** | **Endoplasmic reticulum metallo-peptidase 1** | **60S ribosomal protein L19** |
| **Actin** |  |  |  |  | [29] | [30,31] |  |  |  |  |
| **Neurological disease/ degeneration/ TBI** |  | [32,33] | [34] |  |  | [35,36] |  |  |  |  |
| **Scar formation/ Reactive gliosis** |  |  |  |  |  |  |  |  |  |  |
| **Migration/ Motility/ Chemotaxis** |  |  |  | [37] | [38] |  |  |  |  |  |
| **Proliferation/ Differentiation/ Cell death/ Survival** |  |  | [39,40] | [41] |  | [30,42,43] |  |  |  |  |
| **Engulfment/ Degradation** |  |  |  |  |  |  |  |  |  |  |
| **Neurite/ Growth cones** |  |  | [44] |  | [45] | [46] |  |  |  |  |
| **ER/Golgi/ Secretion/ Energy metabolism** | [47] |  |  | [48] | [29] | [30,43] |  |  |  |  |
| **Immune response** |  | [33,49] |  |  |  |  | [50] |  |  |  |

| **Accession (_MOUSE)** | **CBPE** | **AT2A2** | **THTR** | **GELS** | **GATM** | **ADDA** | **ODO1** | **HXK1** | **AT1B1** | **LAMP2** |
| --- | --- | --- | --- | --- | --- | --- | --- | --- | --- | --- |
| **Protein description** | **Carboxy-peptidase E** | **Sarcoplasmic/ endoplasmic reticulum calcium ATPase 2** | **Thiosulfate sulfur-transferase** | **Gelsolin** | **Glycine amidino-transferase, mitochondrial** | **Alpha-adducin** | **2-oxoglutarate dehydro-genase, mito-chondrial** | **Hexokinase-1** | **Sodium/ potassium-transporting ATPase subunit beta-1** | **Lysosome-associated membrane glycoprotein 2** |
| **Actin** |  |  |  | [51,52,53] |  | [45,54] |  |  |  |  |
| **Neurological disease/ degeneration/ TBI** | [55] |  |  | [56,57] | [58] |  | [59,60] | [61] |  | [56,62,63,64] |
| **Scar formation/ Reactive gliosis** | [55] |  |  |  |  |  |  |  |  |  |
| **Migration/ Motility/ Chemotaxis** |  |  |  | [51,52,65] |  |  |  |  |  |  |
| **Proliferation/ Differentiation/ Cell death/ Survival** | [55] | [66] |  | [57,65] | [58] |  | [60] | [61,67,68] |  |  |
| **Engulfment/ Degradation** |  | [66] |  | [53] |  |  |  |  |  | [62,63,64,69] |
| **Neurite/ Growth cones** |  |  |  | [70] |  |  |  |  |  |  |
| **ER/Golgi/ Secretion/ Energy metabolism** | [29,55] | [20,66,71,72] |  | [57,65] | [58] | [45] | [59,73] | [61,68] |  |  |
| **Immune response** |  |  |  | [51] |  |  |  |  |  | [62,64] |

| **Accession (_MOUSE)** | **CN37** | **FRIL1** | **IF4A3** | **RL18A** | **VA0D1** | **NIPS2** | **TMX2** | **DDX5** | **RUVB1** | **UGPA** |
| --- | --- | --- | --- | --- | --- | --- | --- | --- | --- | --- |
| **Protein description** | **2',3'-cyclic-nucleotide 3'-phospho-diesterase** | **Ferritin light chain 1** | **Eukaryotic initiation factor 4A-III** | **60S ribosomal protein L18a** | **V-type proton ATPase subunit d 1** | **Protein NipSnap homolog 2** | **Thioredoxin-related transmembrane protein 2** | **Probable ATP-dependent RNA helicase DDX5** | **RuvB-like 1** | **UTP--glucose-1-phosphate uridylyl-transferase** |
| **Actin** |  |  |  |  |  |  |  |  |  |  |
| **Neurological disease/ degeneration/ TBI** | [74] | [74,75,76] |  |  |  |  |  | [77] |  |  |
| **Scar formation/ Reactive gliosis** |  |  |  |  |  |  |  |  |  |  |
| **Migration/ Motility/ Chemotaxis** |  |  |  |  |  |  |  |  |  |  |
| **Proliferation/ Differentiation/ Cell death/ Survival** | [78] |  |  |  |  |  | [79] |  |  |  |
| **Engulfment/ Degradation** |  |  |  |  | [80] |  |  |  |  |  |
| **Neurite/ Growth cones** |  |  |  |  |  |  |  |  |  |  |
| **ER/Golgi/ Secretion/ Energy metabolism** |  | [75] |  |  | [80] |  | [79,81] |  |  |  |
| **Immune response** |  |  |  |  |  |  | [79] |  | [50] |  |

| **Accession (_MOUSE)** | **ATP5H** | **RS3** | **NUCL** | **NDUA6** | **RS2** | **ADA28** |
| --- | --- | --- | --- | --- | --- | --- |
| **Protein description** | **ATP synthase subunit d, mitochondrial** | **40S ribosomal protein S3** | **Nucleolin** | **NADH dehydrogenase [ubiquinone] 1 alpha subcomplex subunit 6** | **40S ribosomal protein S2** | **Disintegrin and metalloproteinase domain-containing protein 28** |
| **Actin** |  |  |  |  |  |  |
| **Neurological disease/ degeneration/ TBI** |  |  | [82,83] |  |  |  |
| **Scar formation/ Reactive gliosis** |  |  |  |  |  |  |
| **Migration/ Motility/ Chemotaxis** |  |  |  |  |  |  |
| **Proliferation/ Differentiation/ Cell death/ Survival** |  | [84] |  |  |  |  |
| **Engulfment/ Degradation** |  |  |  |  |  |  |
| **Neurite/ Growth cones** |  |  |  |  |  |  |
| **ER/Golgi/ Secretion/ Energy metabolism** |  |  | [83,85] |  |  | [86] |
| **Immune response** |  |  |  |  |  | [86] |

1. Jaglin XH, Poirier K, Saillour Y, Buhler E, Tian G, et al. (2009) Mutations in the beta-tubulin gene TUBB2B result in asymmetrical polymicrogyria. Nat Genet 41: 746-752.

2. Tischfield MA, Cederquist GY, Gupta ML, Jr., Engle EC (2011) Phenotypic spectrum of the tubulin-related disorders and functional implications of disease-causing mutations. Curr Opin Genet Dev 21: 286-294.

3. Nagai Y, Fujiki M, Inoue R, Uchida S, Abe T, et al. (2005) Neuroprotective effect of geranylgeranylacetone, a noninvasive heat shock protein inducer, on cerebral infarction in rats. Neurosci Lett 374: 183-188.

4. Charrier E, Reibel S, Rogemond V, Aguera M, Thomasset N, et al. (2003) Collapsin response mediator proteins (CRMPs): involvement in nervous system development and adult neurodegenerative disorders. Mol Neurobiol 28: 51-64.

5. Kurnellas MP, Li H, Jain MR, Giraud SN, Nicot AB, et al. (2010) Reduced expression of plasma membrane calcium ATPase 2 and collapsin response mediator protein 1 promotes death of spinal cord neurons. Cell Death Differ 17: 1501-1510.

6. Cains S, Shepherd A, Nabiuni M, Owen-Lynch PJ, Miyan J (2009) Addressing a folate imbalance in fetal cerebrospinal fluid can decrease the incidence of congenital hydrocephalus. J Neuropathol Exp Neurol 68: 404-416.

7. Allen NJ, Rossi DJ, Attwell D (2004) Sequential release of GABA by exocytosis and reversed uptake leads to neuronal swelling in simulated ischemia of hippocampal slices. J Neurosci 24: 3837-3849.

8. Gosselin RD, Bebber D, Decosterd I (2010) Upregulation of the GABA transporter GAT-1 in the gracile nucleus in the spared nerve injury model of neuropathic pain. Neurosci Lett 480: 132-137.

9. Errington AC, Cope DW, Crunelli V (2011) Augmentation of Tonic GABA(A) Inhibition in Absence Epilepsy: Therapeutic Value of Inverse Agonists at Extrasynaptic GABA(A) Receptors. Adv Pharmacol Sci 2011: 790590.

10. Uribe V (2010) The beta-tubulin gene TUBB2B is involved in a large spectrum of neuronal migration disorders. Clin Genet 77: 34-35.

11. Yamashita N, Uchida Y, Ohshima T, Hirai S, Nakamura F, et al. (2006) Collapsin response mediator protein 1 mediates reelin signaling in cortical neuronal migration. J Neurosci 26: 13357-13362.

12. Daugaard M, Kirkegaard-Sorensen T, Ostenfeld MS, Aaboe M, Hoyer-Hansen M, et al. (2007) Lens epithelium-derived growth factor is an Hsp70-2 regulated guardian of lysosomal stability in human cancer. Cancer Res 67: 2559-2567.

13. Vydra N, Winiarski B, Rak-Raszewska A, Piglowski W, Mazurek A, et al. (2009) The expression pattern of the 70-kDa heat shock protein Hspa2 in mouse tissues. Histochem Cell Biol 132: 319-330.

14. Oleinik NV, Krupenko NI, Priest DG, Krupenko SA (2005) Cancer cells activate p53 in response to 10-formyltetrahydrofolate dehydrogenase expression. Biochem J 391: 503-511.

15. Oleinik NV, Krupenko NI, Krupenko SA (2007) Cooperation between JNK1 and JNK2 in activation of p53 apoptotic pathway. Oncogene 26: 7222-7230.

16. Peri F, Nusslein-Volhard C (2008) Live imaging of neuronal degradation by microglia reveals a role for v0-ATPase a1 in phagosomal fusion in vivo. Cell 133: 916-927.

17. Saw NM, Kang SY, Parsaud L, Han GA, Jiang T, et al. (2011) Vacuolar H(+)-ATPase subunits Voa1 and Voa2 cooperatively regulate secretory vesicle acidification, transmitter uptake, and storage. Mol Biol Cell 22: 3394-3409.

18. Deo RC, Schmidt EF, Elhabazi A, Togashi H, Burley SK, et al. (2004) Structural bases for CRMP function in plexin-dependent semaphorin3A signaling. EMBO J 23: 9-22.

19. Poea-Guyon S, Amar M, Fossier P, Morel N (2006) Alternative splicing controls neuronal expression of v-ATPase subunit a1 and sorting to nerve terminals. J Biol Chem 281: 17164-17172.

20. Tupling AR, Bombardier E, Vigna C, Quadrilatero J, Fu M (2008) Interaction between Hsp70 and the SR Ca2+ pump: a potential mechanism for cytoprotection in heart and skeletal muscle. Appl Physiol Nutr Metab 33: 1023-1032.

21. Malchus N, Weiss M (2010) Anomalous diffusion reports on the interaction of misfolded proteins with the quality control machinery in the endoplasmic reticulum. Biophys J 99: 1321-1328.

22. Yamashita N, Morita A, Uchida Y, Nakamura F, Usui H, et al. (2007) Regulation of spine development by semaphorin3A through cyclin-dependent kinase 5 phosphorylation of collapsin response mediator protein 1. J Neurosci 27: 12546-12554.

23. Pelletier MF, Marcil A, Sevigny G, Jakob CA, Tessier DC, et al. (2000) The heterodimeric structure of glucosidase II is required for its activity, solubility, and localization in vivo. Glycobiology 10: 815-827.

24. Morel N, Dedieu JC, Philippe JM (2003) Specific sorting of the a1 isoform of the V-H+ATPase a subunit to nerve terminals where it associates with both synaptic vesicles and the presynaptic plasma membrane. J Cell Sci 116: 4751-4762.

25. Sun-Wada GH, Tabata H, Kawamura N, Aoyama M, Wada Y (2009) Direct recruitment of H+-ATPase from lysosomes for phagosomal acidification. J Cell Sci 122: 2504-2513.

26. Vaz SH, Jorgensen TN, Cristovao-Ferreira S, Duflot S, Ribeiro JA, et al. (2011) Brain-derived neurotrophic factor (BDNF) enhances GABA transport by modulating the trafficking of GABA transporter-1 (GAT-1) from the plasma membrane of rat cortical astrocytes. J Biol Chem 286: 40464-40476.

27. Zhang W, Wearsch PA, Zhu Y, Leonhardt RM, Cresswell P (2011) A role for UDP-glucose glycoprotein glucosyltransferase in expression and quality control of MHC class I molecules. Proc Natl Acad Sci U S A 108: 4956-4961.

28. Dionisio L, Jose De Rosa M, Bouzat C, Esandi Mdel C (2011) An intrinsic GABAergic system in human lymphocytes. Neuropharmacology 60: 513-519.

29. Lou H, Park JJ, Cawley NX, Sarcon A, Sun L, et al. (2010) Carboxypeptidase E cytoplasmic tail mediates localization of synaptic vesicles to the pre-active zone in hypothalamic pre-synaptic terminals. J Neurochem 114: 886-896.

30. Glass-Marmor L, Beitner R (1997) Detachment of glycolytic enzymes from cytoskeleton of melanoma cells induced by calmodulin antagonists. Eur J Pharmacol 328: 241-248.

31. Kusakabe T, Motoki K, Hori K (1997) Mode of interactions of human aldolase isozymes with cytoskeletons. Arch Biochem Biophys 344: 184-193.

32. Garbern JY (2007) Pelizaeus-Merzbacher disease: Genetic and cellular pathogenesis. Cell Mol Life Sci 64: 50-65.

33. Greer JM, Pender MP (2008) Myelin proteolipid protein: an effective autoantigen and target of autoimmunity in multiple sclerosis. J Autoimmun 31: 281-287.

34. Ottervald J, Franzen B, Nilsson K, Andersson LI, Khademi M, et al. (2010) Multiple sclerosis: Identification and clinical evaluation of novel CSF biomarkers. J Proteomics 73: 1117-1132.

35. Mor F, Izak M, Cohen IR (2005) Identification of aldolase as a target antigen in Alzheimer's disease. J Immunol 175: 3439-3445.

36. Reed TT, Pierce WM, Jr., Turner DM, Markesbery WR, Butterfield DA (2009) Proteomic identification of nitrated brain proteins in early Alzheimer's disease inferior parietal lobule. J Cell Mol Med 13: 2019-2029.

37. Baumann F, Leukel P, Doerfelt A, Beier CP, Dettmer K, et al. (2009) Lactate promotes glioma migration by TGF-beta2-dependent regulation of matrix metalloproteinase-2. Neuro Oncol 11: 368-380.

38. Chen CL, Hsieh YT, Chen HC (2007) Phosphorylation of adducin by protein kinase Cdelta promotes cell motility. J Cell Sci 120: 1157-1167.

39. Lamprianou S, Chatzopoulou E, Thomas JL, Bouyain S, Harroch S (2011) A complex between contactin-1 and the protein tyrosine phosphatase PTPRZ controls the development of oligodendrocyte precursor cells. Proc Natl Acad Sci U S A 108: 17498-17503.

40. Xenaki D, Martin IB, Yoshida L, Ohyama K, Gennarini G, et al. (2011) F3/contactin and TAG1 play antagonistic roles in the regulation of sonic hedgehog-induced cerebellar granule neuron progenitor proliferation. Development 138: 519-529.

41. Marin-Hernandez A, Gallardo-Perez JC, Ralph SJ, Rodriguez-Enriquez S, Moreno-Sanchez R (2009) HIF-1alpha modulates energy metabolism in cancer cells by inducing over-expression of specific glycolytic isoforms. Mini Rev Med Chem 9: 1084-1101.

42. Lupo A, Costanzo P, De Rosa M, Russo T, Salvatore F, et al. (1994) Growth-arrested dependence of aldolase A L-type mRNA expression in rodent cell lines. Exp Cell Res 213: 359-364.

43. Schafer D, Hamm-Kunzelmann B, Brand K (1997) Glucose regulates the promoter activity of aldolase A and pyruvate kinase M2 via dephosphorylation of Sp1. FEBS Lett 417: 325-328.

44. Dityatev A, Bukalo O, Schachner M (2008) Modulation of synaptic transmission and plasticity by cell adhesion and repulsion molecules. Neuron Glia Biol 4: 197-209.

45. Stevens RJ, Littleton JT (2011) Synaptic growth: dancing with adducin. Curr Biol 21: R402-405.

46. Blackmore MG, Moore DL, Smith RP, Goldberg JL, Bixby JL, et al. (2010) High content screening of cortical neurons identifies novel regulators of axon growth. Mol Cell Neurosci 44: 43-54.

47. Hiltunen JK (1991) Peroxisomes and beta-oxidation of long-chain unsaturated carboxylic acids. Scand J Clin Lab Invest Suppl 204: 33-46.

48. Granchi C, Bertini S, Macchia M, Minutolo F (2010) Inhibitors of lactate dehydrogenase isoforms and their therapeutic potentials. Curr Med Chem 17: 672-697.

49. Kuchroo VK, Anderson AC, Waldner H, Munder M, Bettelli E, et al. (2002) T cell response in experimental autoimmune encephalomyelitis (EAE): role of self and cross-reactive antigens in shaping, tuning, and regulating the autopathogenic T cell repertoire. Annu Rev Immunol 20: 101-123.

50. Castorena CM, Mackrell JG, Bogan JS, Kanzaki M, Cartee GD (2011) Clustering of GLUT4, TUG and RUVBL2 Protein Levels Correlate with Myosin Heavy Chain Isoform Pattern in Skeletal Muscles, but AS160 and TBC1D1 Levels Do Not. J Appl Physiol.

51. Witke W, Sharpe AH, Hartwig JH, Azuma T, Stossel TP, et al. (1995) Hemostatic, inflammatory, and fibroblast responses are blunted in mice lacking gelsolin. Cell 81: 41-51.

52. Rombouts K, Knittel T, Machesky L, Braet F, Wielant A, et al. (2002) Actin filament formation, reorganization and migration are impaired in hepatic stellate cells under influence of trichostatin A, a histone deacetylase inhibitor. J Hepatol 37: 788-796.

53. Groves E, Dart AE, Covarelli V, Caron E (2008) Molecular mechanisms of phagocytic uptake in mammalian cells. Cell Mol Life Sci 65: 1957-1976.

54. Seidel B, Zuschratter W, Wex H, Garner CC, Gundelfinger ED (1995) Spatial and sub-cellular localization of the membrane cytoskeleton-associated protein alpha-adducin in the rat brain. Brain Res 700: 13-24.

55. Koshimizu H, Senatorov V, Loh YP, Gozes I (2009) Neuroprotective protein and carboxypeptidase E. J Mol Neurosci 39: 1-8.

56. Natale JE, Ahmed F, Cernak I, Stoica B, Faden AI (2003) Gene expression profile changes are commonly modulated across models and species after traumatic brain injury. J Neurotrauma 20: 907-927.

57. Chauhan V, Ji L, Chauhan A (2008) Anti-amyloidogenic, anti-oxidant and anti-apoptotic role of gelsolin in Alzheimer's disease. Biogerontology 9: 381-389.

58. Beard E, Braissant O (2010) Synthesis and transport of creatine in the CNS: importance for cerebral functions. J Neurochem 115: 297-313.

59. Adam-Vizi V (2005) Production of reactive oxygen species in brain mitochondria: contribution by electron transport chain and non-electron transport chain sources. Antioxid Redox Signal 7: 1140-1149.

60. Calingasan NY, Ho DJ, Wille EJ, Campagna MV, Ruan J, et al. (2008) Influence of mitochondrial enzyme deficiency on adult neurogenesis in mouse models of neurodegenerative diseases. Neuroscience 153: 986-996.

61. Regenold WT, Pratt M, Nekkalapu S, Shapiro PS, Kristian T, et al. (2012) Mitochondrial detachment of hexokinase 1 in mood and psychotic disorders: implications for brain energy metabolism and neurotrophic signaling. J Psychiatr Res 46: 95-104.

62. Ruivo R, Anne C, Sagne C, Gasnier B (2009) Molecular and cellular basis of lysosomal transmembrane protein dysfunction. Biochim Biophys Acta 1793: 636-649.

63. Qin AP, Liu CF, Qin YY, Hong LZ, Xu M, et al. (2010) Autophagy was activated in injured astrocytes and mildly decreased cell survival following glucose and oxygen deprivation and focal cerebral ischemia. Autophagy 6: 738-753.

64. Wu G, Wang X, Feng X, Zhang A, Li J, et al. (2011) Altered expression of autophagic genes in the peripheral leukocytes of patients with sporadic Parkinson's disease. Brain Res 1394: 105-111.

65. Kronenberg G, Gertz K, Baldinger T, Kirste I, Eckart S, et al. (2010) Impact of actin filament stabilization on adult hippocampal and olfactory bulb neurogenesis. J Neurosci 30: 3419-3431.

66. Law BY, Wang M, Ma DL, Al-Mousa F, Michelangeli F, et al. (2010) Alisol B, a novel inhibitor of the sarcoplasmic/endoplasmic reticulum Ca(2+) ATPase pump, induces autophagy, endoplasmic reticulum stress, and apoptosis. Mol Cancer Ther 9: 718-730.

67. Rathmell JC, Fox CJ, Plas DR, Hammerman PS, Cinalli RM, et al. (2003) Akt-directed glucose metabolism can prevent Bax conformation change and promote growth factor-independent survival. Mol Cell Biol 23: 7315-7328.

68. Saraiva LM, Seixas da Silva GS, Galina A, da-Silva WS, Klein WL, et al. (2010) Amyloid-beta triggers the release of neuronal hexokinase 1 from mitochondria. PLoS One 5: e15230.

69. Huynh KK, Eskelinen EL, Scott CC, Malevanets A, Saftig P, et al. (2007) LAMP proteins are required for fusion of lysosomes with phagosomes. EMBO J 26: 313-324.

70. Tanaka J, Kira M, Sobue K (1993) Gelsolin is localized in neuronal growth cones. Brain Res Dev Brain Res 76: 268-271.

71. Pani B, Singh BB (2008) Darier's disease: a calcium-signaling perspective. Cell Mol Life Sci 65: 205-211.

72. Bigelow DJ (2009) Nitrotyrosine-modified SERCA2: a cellular sensor of reactive nitrogen species. Pflugers Arch 457: 701-710.

73. Marcaggi P, Coles JA (2001) Ammonium in nervous tissue: transport across cell membranes, fluxes from neurons to glial cells, and role in signalling. Prog Neurobiol 64: 157-183.

74. Connor JR, Ponnuru P, Lee BY, Podskalny GD, Alam S, et al. (2011) Postmortem and imaging based analyses reveal CNS decreased myelination in restless legs syndrome. Sleep Med 12: 614-619.

75. Barbeito AG, Garringer HJ, Baraibar MA, Gao X, Arredondo M, et al. (2009) Abnormal iron metabolism and oxidative stress in mice expressing a mutant form of the ferritin light polypeptide gene. J Neurochem 109: 1067-1078.

76. Luscieti S, Santambrogio P, Langlois d'Estaintot B, Granier T, Cozzi A, et al. (2010) Mutant ferritin L-chains that cause neurodegeneration act in a dominant-negative manner to reduce ferritin iron incorporation. J Biol Chem 285: 11948-11957.

77. Kar A, Fushimi K, Zhou X, Ray P, Shi C, et al. (2011) RNA helicase p68 (DDX5) regulates tau exon 10 splicing by modulating a stem-loop structure at the 5' splice site. Mol Cell Biol 31: 1812-1821.

78. Scherer SS, Braun PE, Grinspan J, Collarini E, Wang DY, et al. (1994) Differential regulation of the 2',3'-cyclic nucleotide 3'-phosphodiesterase gene during oligodendrocyte development. Neuron 12: 1363-1375.

79. Masutani H, Ueda S, Yodoi J (2005) The thioredoxin system in retroviral infection and apoptosis. Cell Death Differ 12 Suppl 1: 991-998.

80. Saroussi S, Nelson N (2009) The little we know on the structure and machinery of V-ATPase. J Exp Biol 212: 1604-1610.

81. Meng X, Zhang C, Chen J, Peng S, Cao Y, et al. (2003) Cloning and identification of a novel cDNA coding thioredoxin-related transmembrane protein 2. Biochem Genet 41: 99-106.

82. Reiser G, Bernstein HG (2004) Altered expression of protein p42IP4/centaurin-alpha 1 in Alzheimer's disease brains and possible interaction of p42IP4 with nucleolin. Neuroreport 15: 147-148.

83. Caudle WM, Kitsou E, Li J, Bradner J, Zhang J (2009) A role for a novel protein, nucleolin, in Parkinson's disease. Neurosci Lett 459: 11-15.

84. Kim TS, Jang CY, Kim HD, Lee JY, Ahn BY, et al. (2006) Interaction of Hsp90 with ribosomal proteins protects from ubiquitination and proteasome-dependent degradation. Mol Biol Cell 17: 824-833.

85. Kibbey MC, Johnson B, Petryshyn R, Jucker M, Kleinman HK (1995) A 110-kD nuclear shuttling protein, nucleolin, binds to the neurite-promoting IKVAV site of laminin-1. J Neurosci Res 42: 314-322.

86. Fourie AM, Coles F, Moreno V, Karlsson L (2003) Catalytic activity of ADAM8, ADAM15, and MDC-L (ADAM28) on synthetic peptide substrates and in ectodomain cleavage of CD23. J Biol Chem 278: 30469-30477.
